# Mucous Fistula Refeeding in Newborns: Why, When, How, and Where? Insights from a Systematic Review

**DOI:** 10.3390/nu17152490

**Published:** 2025-07-30

**Authors:** Layla Musleh, Ilaria Cozzi, Anteo Di Napoli, Fabio Fusaro

**Affiliations:** 1 Department of Public Health and Infectious Disease, Sapienza University of Rome, 00185 Rome, Italy; layla.musleh@uniroma1.it; 2 Department of Pediatric Surgery, San Camillo-Forlanini Hospital, 00152 Rome, Italy; 3 Department of Epidemiology, Lazio Regional Health Service, Local Health Unit 1, 00147 Rome, Italy; i.cozzi@deplazio.it; 4 Epidemiology Unit, National Institute for Health, Migration and Poverty (INMP), 00153 Rome, Italy; anteo.dinapoli@inmp.it; 5 Surgical Rehabilitation Intestinal Failure Unit, Bambino Gesù Children’s Hospital, IRCCS, 00163 Rome, Italy

**Keywords:** mucous fistula refeeding, neonatal enterostomy, parenteral nutrition, intestinal adaptation, clinical outcomes

## Abstract

**Background/Objectives**: Infants with high-output enterostomies often require prolonged parenteral nutrition (PN), increasing risks of infections, liver dysfunction, and impaired growth. Mucous fistula refeeding (MFR) is proposed to enhance intestinal adaptation, weight gain, and distal bowel maturation. This systematic review and meta-analysis assessed its effectiveness, safety, and technical aspects. **Methods**: Following PRISMA guidelines, studies reporting MFR-related outcomes were included without data or language restrictions. Data sources included PubMed, EMBASE, CINAHL, Scopus, Web of Science, Cochrane Library, and UpToDate. Bias risk was assessed using the Joanna Briggs Institute Critical Appraisal Checklist. Meta-analysis employed random- and fixed-effects models, with outcomes reported as odds ratios (ORs) and 95% confidence interval (CI). Primary outcomes assessed were weight gain, PN duration, and complications and statistical comparisons were made between MFR and non-MFR groups. **Results**: Seventeen studies involving 631 infants were included; 482 received MFR and 149 did not. MFR started at 31 postoperative days and lasted for 50 days on average, using varied reinfusion methods, catheter types, and fixation strategies. MFR significantly improved weight gain (4.7 vs. 24.2 g/day, *p* < 0.05) and reduced PN duration (60.3 vs. 95 days, *p* < 0.05). Hospital and NICU stays were also shorter (160 vs. 263 days, *p* < 0.05; 122 vs. 200 days, *p* < 0.05). Cholestasis risk was lower (OR 0.151, 95% CI 0.071–0.319, *p* < 0.0001), while effects on bilirubin levels were inconsistent. Complications included sepsis (3.5%), intestinal perforation (0.83%), hemorrhage (0.62%), with one MFR-related death (0.22%). **Conclusions**: Despite MFR benefits neonatal care, its practices remain heterogeneous. Standardized protocols are required to ensure MFR safety and efficacy.

## 1. Introduction

In neonatal surgery, conditions such as necrotizing enterocolitis, meconium peritonitis, and bowel atresia may require a temporary enterostomy, consisting of a proximal stoma and a distal mucous fistula (MF) [1,2,3,4]. While enterostomies are life-saving procedures, they carry a complication rate of 24–68%, including prolapse, stoma stenosis, peristomal skin breakdown, wound infection, and obstruction. [5,6,7,8,9]. High-output enterostomies can result in significant fluid, nutrient, and electrolyte losses, often requiring prolonged parenteral nutrition (PN). Consequently, this increases the risk of PN-related complications, extends hospitalization, raises healthcare costs, and reduces quality of life [10]. Although temporary stomas should be maintained only for the time required for recovery, the optimal timing for bowel continuity restoration (BCR) in neonates remains debated [11]. Many infants present comorbidities that delay surgery, exposing them to risks of transient type 2 intestinal failure and prolonged PN, as highlighted by European Society for Clinical Nutrition and Metabolism (ESPEN) [12,13,14]. MF refeeding (MFR) has been proposed as a strategy to enhance the absorptive function of the excluded distal bowel. First described in the 1980s as “entero-enteric re-circulation,” MFR involves reinfusing chyme from the proximal stoma into the efferent bowel loop via an extracorporeal bypass to restore physiological transit and promote bowel adaptation [15]. Despite its increasing adoption, the literature on MFR remains limited, particularly concerning its indications, methodologies, and clinical outcomes. This systematic review aims to summarize the indications and methods of MFR, assess its impact on nutritional biomarkers and bowel maturation, and identify its limitations. By clarifying its clinical application, this review seeks to provide insights that may guide future research and clinical practice.

## 2. Materials and Methods

### 2.1. Search Strategy

No approval from an ethics committee or institutional review board was required. This review follows the Preferred Reporting Items for Systematic Reviews and Meta-Analyses (PRISMA) statement (https://www.prisma-statement.org/), with a protocol registered in PROSPERO (CRD42024533070). A systematic search was conducted across PubMed, MEDLINE, CINAHL, Scopus, the Web of Science, the Cochrane Library, and UpToDate from inception to April 2023. There was no restriction on language or publication date. Non-English studies were assessed and included if sufficient data could be extracted, either directly or through translation. The search combined free-text terms related to “ostomy” or “fistula” with “refeeding” using the AND operator (Supplementary Materials Table S1). Reference lists of relevant articles and systematic reviews were also screened.

### 2.2. Inclusion and Exclusion Criteria—Selection Criteria

Observational studies reporting outcomes in infants with a double enterostomy, formed within 28 days after birth or at a corrected gestational age of 42 weeks, who received MFR were included.

To avoid data duplication, only the most recent and comprehensive study of each cohort was considered. Excluded articles included those reporting data on fewer than five patients, those focusing solely on refeeding techniques, as well abstracts, commentaries, case reports, letters, conference abstracts, and non-peer-reviewed articles. Systematic reviews were also excluded to ensure original research was analyzed.

### 2.3. Main Outcomes

Outcome measures were categorized as follows:

- Reinfusion methods: collection and reinfusion modalities, catheter types, safety measures, and personnel involved in performing MFR;
- Nutritional biomarkers: cholestasis, catheter-related sepsis, BCR time, mean peak bilirubin, PN duration, hospital and Neonatal Intensive Care Unit (NICU) stay, weight gain before and during MFR, and distal bowel maturation indicators (size discrepancy reduction, anastomosis complications);
- Limitations: technical concerns and adverse events.

### 2.4. Quality Assessment

Study quality was assessed using the Joanna Briggs Institute (JBI) Critical AppraisalChecklist (https://jbi.global/critical-appraisal-tools, accessed on 14 January 2025). Some studies initially classified as case-control were reclassified as cohort studies based on exposure rather than disease status [16]. Studies were rated for risk of bias (RoB) as high (≤49%), moderate (50–69%), or low (≥70%). Two independent reviewers conducted assessments (Supplementary Materials Table S2).

### 2.5. Data Extraction

Search results were uploaded to Rayyan (https://www.rayyan.ai/), a web-based platform designed to facilitate systematic review screening. Two independent researchers (L.M. and I.C.) reviewed studies, with discrepancies resolved through discussion and arbitration (F.F.). Data extraction was conducted independently by two authors and validated by a third (A.D.N.). Extracted parameters included study details, demographics, reasons for stoma formation, stoma site, MFR initiation criteria, equipment used, and outcomes measures.

### 2.6. Data Reporting and Analysis

Meta-analysis was performed on studies reporting data in both MFR and non-MFR groups. If meta-analysis was not feasible, a narrative synthesis was provided. Data were extracted as mean ± standard deviation (SD) or median (interquartile range, IQR). When necessary, SDs were estimated using Wan et al.’s method [17].

Statistical methods included:

- Random-effects model: accounted for inter-study variability using Hedges’s g for continuous outcomes and odds ratios (ORs) for categorical outcomes;
- Fixed-effects model: assumed a common effect size across studies;
- Chi-square test: assessed categorical variable distributions between MFR and non-MFR groups;
- Two-sample *t*-test with unequal variances: compared mean differences between groups.

Significance was set at *p* < 0.05 with 95% confidence intervals (CI). Meta-analysis was performed when data were available for both MFR and comparator groups. Statistical analyses were conducted using Stata Version 18 (StataCorp LLC, College Station, TX, USA). Using both random- and fixed-effects models allowed for a more comprehensive evaluation, enhancing the robustness and generalizability of findings.

## 3. Results

### 3.1. Search Outcomes

**Figure 1:**
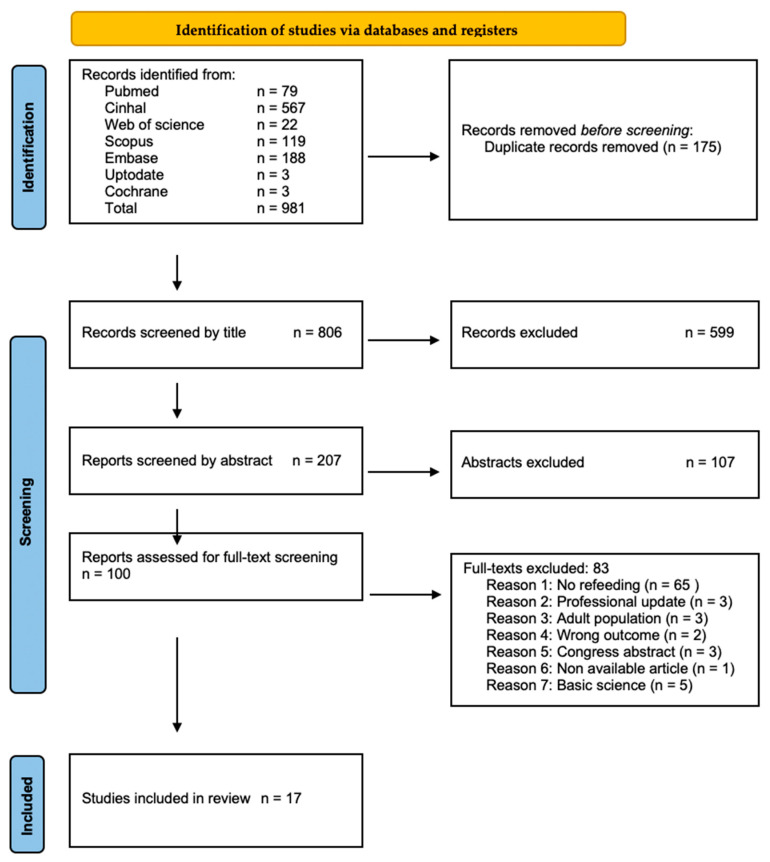
Study Selection Process. Preferred Reporting Items for Systematic Reviews and Meta-Analyses (PRISMA) flow chart (reproduced from Ref. [34], https://www.prisma-statement.org/).

A total of 17 articles were included (Figure 1) [1,18,19,20,21,22,23,24,25,26,27,28,29,30,31,32,33]. These were largely retrospective studies [1,18,19,20,21,22,23,24,26,27,28,29,30,31,32], with one ambispective study [25] and one randomized controlled trial (RCT) [33]. The studies reported MFR experiences as case series [1,19,22,23,24,26,29] and cohort studies [18,20,21,25,27,28,30,31,32]. The articles encompassed data from 631 patients (Supplementary Materials Table S3). Among these, 482 were neonates receiving chymus MFR (*n* = 456, 72.3%) [20,21,28,30,31,32,33] or normal saline MFR (*n* = 26, 4.1%) [25], and 149 did not receive any treatment. The group receiving normal saline was included within the MFR cohort considering the mechanical effects in an otherwise unoccupied intestinal segment.

### 3.2. Intervention Descriptors

Among the patients, 323 were males (51.2%). Mean gestational age was 30.7 vs. 28.4 weeks (*p* < 0.001), and birth weight was 1630.3 vs. 1347.1 *g* (*p* < 0.001) in the MFR and non-treated group, respectively. Mean age at stoma formation was 15.0 vs. 20.6 days (*p* < 0.05) in the MFR and non-treated groups. Mean time and weight at BCR were 86.1 vs. 71.9 days (*p* < 0.05) and 2949.9 and 1788.0 *g* (*p* < 0.05), respectively.

Comparing patients who received MFR and those who did not, the most common surgical indications were necrotizing enterocolitis (60.4% vs. 8.1%, *p* < 0.07), small bowel atresia (13.9% vs. 8.1%, *p* = 0.06), meconium ileus (11.8% vs. 2.7%, *p* = 0.08), spontaneous intestinal perforation (10.9% vs. 1.3%, *p* < 0.001), malrotation with or without midgut volvulus (3.1% vs. 5.4%, *p* = 0.20), and abdominal wall defects (0.6% vs. 5.4%, *p* < 0.001) (Supplementary Materials Figure S1).

The proximal stoma anatomical site was jejunum in 9.9% vs. 4.7% (*p* = 0.07) and ileum in 36% vs. 52.3% (*p* < 0.001) (Supplementary Materials Figure S2). Mean residual bowel length was 62.2 cm vs. 55.9 cm (*p* < 0.05), and the ileocecal valve was resected in 27.7% of cases receiving MFR vs. 8.0% in non-MFR cases (*p* < 0.001).

MFR started on average on the 31st post-operative day and was administered for a mean duration of 50 days. Mean daily weight gain before and during MFR was 4.7 vs. 24.2 *g* (*p* < 0.05). PN mean duration was 60.3 vs. 95 days (*p* < 0.05) in MFR and non-MFR groups. Total hospital stay was 160 vs. 263 days (*p* < 0.05), and NICU stay was 122 vs. 200 days (*p* < 0.05) in the two groups.

### 3.3. First Outcome—Methods of Mucous Fistula Refeeding

Various MFR methods have been outlined (Supplementary Materials Table S4). Indications included: high output stoma (18%) [19,24,33], growth retardation (18%) [20,23,24], presence of enterostomy (12%) [22,26], surgeon’s clinical decision (12%) [28,31], and stimulation of the distal bowel (6%) [25]. MFR was primarily administered in the hospital setting by the surgical team (35%) [19,22,28,30,32,33] and/or nursing staff (23%) [18,19,22,32,33], with three authors reporting home parental administration (18%) [24,26,29]. Reinfused substances included proximal stoma effluent in 65% of cases [1,18,19,20,21,23,24,25,27,28,31], normal saline in 12% [22,25]; chyme mixed (18%) the with semi-elemental milk [26] or normal saline [29,32], and priming MFR with normal saline for three days was reported in one article (6%) [30].

Intestinal content aspiration from the proximal stoma was primarily manual, utilizing Luer lock syringes and/or standard stoma bags (59%) [18,20,22,23,24,26,27,29,30,33]. Two authors filtered the proximal stoma output using dry gauze (12%) [20,29]. Collection intervals varied from 3 to 8 h. Refeeding modalities involved MF catheterization using probes of different types and sizes, advanced into the limb for 3–10 cm, and secured variably by inflated balloons, tapes, and dressings. Two articles protected the MF from skin erosion through a skin barrier (12%) [23,24].

Two authors reported placing a catheter in the rectum to enhance the absorptive capacity of the distal bowel [22,26]. Automated methods for infusion, such as syringe or roller pumps, were more commonly employed (59%) [1,20,21,23,24,25,26,27,28,31] rather than manual infusion (35%) [18,22,29,30,32,33]. Continuous (59%) delivery modality was preferred over bolus (29%). Schafer et al. introduced a novel MFR method with a closed system (‘Continuous Extracorporeal Stool Transport–CEST’): an adhesive stoma bag connected to a silicon tube in series, through which proximal stoma output was collected and delivered through a roller pump [25].

### 3.4. Second Outcome—Clinical Effects of Mucous Fistula Refeeding in Neonates

**Figure 2:**
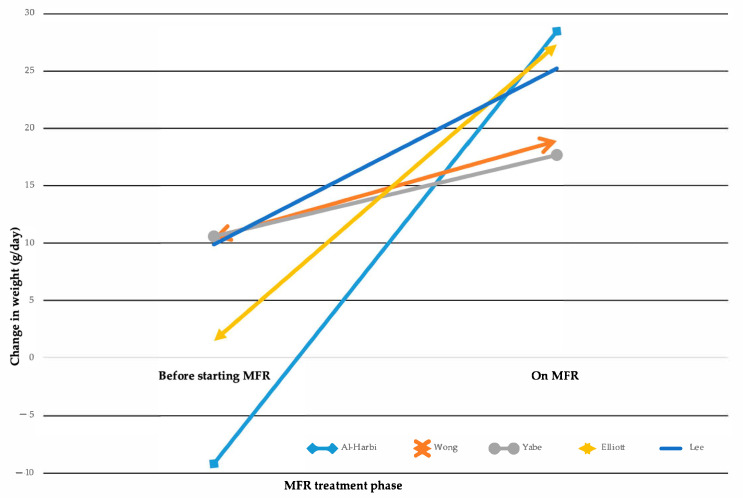
Changes in weight gain while on Mucous Fistula Refeeding (MFR). Multiline graph comparing weight change from the time of ostomy formation to the start of MFR treatment vs. the weight change achieved during MFR treatment, as reported in five studies [1,18,23,24,33].

MFR demonstrates varying degrees of improvement in post-operative nutritional biomarkers (Supplementary Materials Table S6). Analysis of mean weight gain showed significant differences between groups (Figure 2) [1,18,23,24,33].

**Figure 3:**
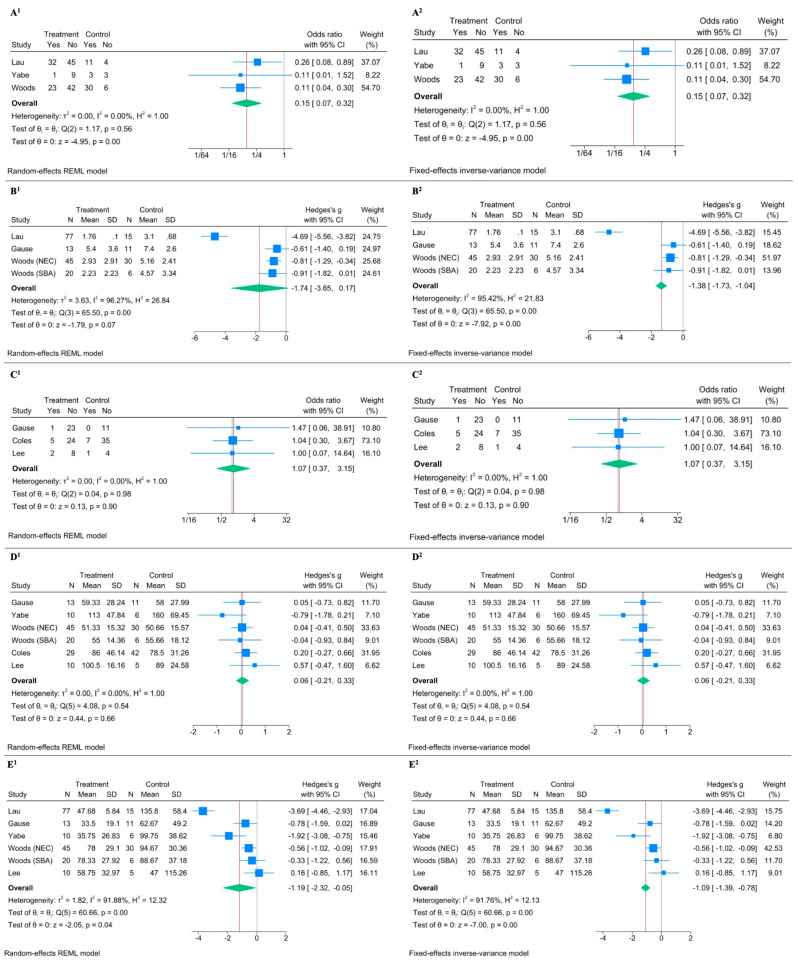
Forest plot illustrating the effect of Mucous Fistula Refeeding (MFR) on selected clinical outcomes. Superscript 1 refers to the random-effects model; superscript 2 to the fixed-effects model. (**A^1^**,**A^2^**) MFR significantly reduces the risk of cholestasis (OR^1^ = 0.15, 95% CI: 0.07–0.32, *p* < 0.0001, *I*^2^ = 0.00%). (**B^1^**,**B^2^**) No significant difference in serum bilirubin peak between MFR and control groups (Hedges’ g^1^ = –1.74, 95% CI: –3.65 to 0.17, *p* = 0.07, *I*^2^ = 96.27%). (**C^1^**,**C^2^**) No difference in risk of sepsis (OR^1^ = 1.07, 95% CI: 0.37–3.15, *p* = 0.90, *I*^2^ = 0.00%). (**D^1^**,**D^2^**) No difference in time to bowel continuity restoration (Hedges’ g^1^ = 0.06, 95% CI: –0.21 to 0.33, *p* = 0.66, *I*^2^ = 0.00%). (**E^1^**,**E^2^**) MFR is associated with a shorter duration of parenteral nutrition (Hedges’ g^1^ = –1.19, 95% CI: –2.32 to –0.05, *p* = 0.04, *I*^2^ = 91.88%) [18,21,28,31,32,33].

Meta-analysis regarding cholestasis showed a significant reduction in the MFR group with an OR of 0.151 with a 95% CI of 0.071 to 0.319, and a *p*-value < 0.0001 in the random-effects model (Figure 3(A^1^)) [18,21,31]. The fixed-effects model provided similar results (Figure 3(A^2^)). The low heterogeneity (*I*^2^ = 0.00%) further supported the consistency of the effect across studies. The effect of MFR on peak bilirubin levels showed heterogeneous results (*I*^2^ = 96.27%) [21,28,31]. While the random-effects model gave an effect size (Hedges’s g) of −1.742 with a 95% CI of −3.651 to 0.166 and a *p*-value of 0.0736 (indicating a non-significant result), the fixed-effects model showed a significant effect (effect size of −1.385 and a 95% CI of −1.728 to −1.042, with a *p*-value < 0.0001) (Figure 3(B^1^,B^2^)). Regarding catheter-related sepsis, the meta-analysis suggested no significant effect: the random-effects model yielded an OR of 1.074 with a 95% CI of 0.366 to 3.153 and a *p*-value of 0.8968, and the fixed-effects model showed similar OR and CI values (Figure 3(C^1^,C^2^)) [28,32,33]. The high *p*-value for heterogeneity (Q = 0.04, *p* = 0.9802) suggested no significant variation among the studies. No significant effect was found on the time for BCR in both random-effects and fixed-effects meta-analysis (Figure 3(D^1^,D^2^)) [18,28,31,32,33]. PN duration analysis suggested a significant reduction in PN duration with MFR, despite significant heterogeneity given by one outlier (*I*^2^ = 91.88%) [18,21,28,31,33]. The random-effects model showed an effect size (Hedges’s g) of −1.187 with a 95% CI of −2.323 to −0.051 and a *p*-value of 0.0405 (Figure 3(E^1^)), and the fixed-effects model confirmed a significant effect size of −1.085 with a 95% CI of −1.389 to −0.781 and a *p*-value of 0.0000 (Figure 3(E^2^)).

Regarding distal bowel maturation indicators, one study showed a protective effect, reducing bowel discrepancy at BCR (OR 0.29, 95%CI 0.08, 1.05, *p* = 0.04) and post-BCR complications, such as anastomotic dehiscence or stenosis (OR 0.11, 95%CI 0.01, 1.07, *p* = 0.03) [21]. One study reported reduced total length of hospital (mean difference −83.6, Std. Err. 24.82, 95%CI −136.60, −30.06, *p* = 0.0043) and NICU stay (mean difference −74.5, Std. Err. 16.50, 95%CI −109.76, −39.24, *p* = 0.0004) in the MFR group [21].

Although funnel plots were generated to assess publication bias for the outcomes, the interpretation was inconclusive according to Cochrane guidelines due to the limited number of studies available (https://training.cochrane.org/handbook/current/chapter-13#section-13-3-5-4, accessed on 14 January 2025). (Supplementary Materials Table S5).

### 3.5. Third Outcome—Adverse Events Related to Mucous Fistula Refeeding

MFR-associated complications were reported in eight studies (47%) (Table 1). Although most included articles mentioned at least one adverse event, a reliable calculation of the overall complication rate was not feasible due to heterogeneous and incomplete reporting. While some studies provided numerical data, many described complications narratively without specifying incidence or total number of affected patients. Where available, complication rates are reported.

The overall mortality rate among MFR patients was 2.08%. However, mortality data for patients for non-MFR patients were not explicitly reported, limiting direct comparison. One death (0.22%) was directly attributed to the procedure, in a complex clinical course involving abdominal compartment syndrome, septic shock, gastrointestinal bleedings, and multiple organ failure [19]. Other reported causes of mortality included respiratory sepsis (4 cases) [1,19], complications following cardiac surgery (1 case) [1], multiorgan failure after stoma closure (2 cases) [19,30], septic cardiorespiratory failure (1 case) [19], and complications related to prematurity (1 case) [29].

Several technical concerns impeded MFR, notably the dislodgement [19,21,28], reflux, or leakage of intestinal contents [19,21,26].

Reported complications included distal stoma prolapse (0.62%) [23,30,33], peristomal skin irritation (0.62%) [23,33], wound infection (0.42%) [23], MF stenosis (0.21%) [30], entero-cutaneous fistula (0.21%) [23], and bowel distention [26,33]. Rare but life-threatening events included bacterial translocation and sepsis (3.5%) [1,19,33], intestinal perforation (0.83%) [19,33], and hemorrhage (0.62%) [19,30].

Where data were available, the overall incidence of MFR-related complications of any severity ranged from approximately 10% to 25%. Minor adverse events such as stoma dislodgement, local irritation, or backflow were frequently mentioned, although often without numerical detail.

## 4. Discussion

Our findings need to be interpreted within the complex anatomical and physiological setting created by a double enterostomy, which divides the bowel into two non-communicating segments: a proximal loop with limited digestive and absorptive capacity, and a distal limb deprived of enteric flow and secretions. Building on the early work by Puppala et al. in the 1980s, who first described a continuous infusion of stoma output via glucose drip, MFR has since evolved into a more structured practice [15].

In this context, MFR offers a potential means to restore intestinal function by reintroducing proximal chyme into the distal bowel, thereby preserving bile salt circulation, stimulating mucosal maturation, and potentially improving post-reversal outcomes, although this has not been directly demonstrated in the available clinical studies.

However, despite promising evidence, its clinical adoption remains inconsistent across centers, contributing to heterogeneity in the reported outcomes.

### 4.1. Effects of MFR

This review examined MFR use in neonates, analyzing 17 studies. MFR demonstrated positive clinical outcomes with rare serious adverse events (Table 1). Our meta-analysis indicates that MFR enhances intestinal absorption, improves nutritional status, mitigates cholestasis, and reduces PN dependence [1,18,21,23,24,31,33]. Histopathology studies reveal that MFR maintains mucosal thickness and villous structure, contrasting with the mucosal atrophy of the non-MFR group (14). Although mean peak bilirubin levels showed no significant difference between groups, our meta-analysis demonstrated an 85% lower risk of PNALD in MFR patients. The MUC-FIRE multicenter trial aims to evaluate whether MFR can expedite the transition to full enteral feeding, potentially reducing PN dependence and hospital stay [35].

### 4.2. Complications Associated with MFR

Despite its benefits, MFR poses challenges. Adverse events may be linked to underlying conditions or procedural factors due to lack of specific knowledge, training, or established protocols. Complications range from mild issues like peristomal skin irritation [23,33] and localized wound infections [23] to more serious complications, including sepsis [1,19,33], intestinal perforation [19,33], and hemorrhage [19,30]. Sepsis, the most frequent severe event, likely results from translocation and altered permeability, necessitating vigilant monitoring. The proximal intestinal microbiome remains poorly understood, and in vivo culture challenges further complicate microbiological assessments. Yabe et al. reported no pathogenic bacteria in proximal stoma output collected after 3 h, [18], whereas other studies suggest microbial alterations as early as 1.5 h [33,36]. To counter stagnation, Schäfer et al. proposed an extracorporeal stool transport system in 1997 [25]. Less frequently, infections may be linked to mechanical complications such as perforation, leading to peritonitis, sepsis, or mucosal bleeding [19]. In our analysis, severe complications occurred in fewer than 1% of patients, but the overall mortality rate of 2.19% highlights the need for caution. In addition to clinical risks, MFR poses practical and emotional challenges, particularly when performed outside the hospital setting. While most studies reported MFR administration by medical or nursing staff in NICU, three authors in our review described successful implementation at home by parents or caregivers [24,26,29]. Although feasible, this approach requires meticulous caregiver education, aseptic technique, and close monitoring. These factors underscore the need for structured discharge planning, psychosocial support, and formal training pathways to ensure the safety and sustainability of home-based MFR.

Further research is needed to understand mechanisms driving infective complications and improving MFR safety.

### 4.3. Cost Considerations

PN is costly, with European estimates placing the daily cost of one compounded PN bag for neonates at approximately €55.16, and NICU charges reaching $4000 per day in the United States [37,38,39]. Although the economic impact of reduced PN use is not extensively analyzed, shortening PN dependence and hospital stays through MFR could yield substantial cost savings.

### 4.4. Challenges in Interpreting Study Outcomes

Meta-analysis requires addressing heterogeneity—variations in study outcomes that cannot be attributed solely to chance. Heterogeneity can arise from differences in study populations, interventions, outcome measures, and methodologies, which significantly affect the reliability of pooled estimates. When heterogeneity is high, as indicated by an *I*^2^ statistic above 50%, it suggests that the studies may not be estimating the same underlying effect. In such cases, a random-effects model is preferable, as it accounts for both within-study and between-study variability. Conversely, a fixed-effects model, which assumes that all studies estimate the same true effect size, is more appropriate when heterogeneity is low.

A key limitation of this meta-analysis is the presence of baseline imbalances between the MFR and non-MFR groups in several included studies, including differences in birth weight, residual bowel length, and underlying diagnosis (e.g., NEC prevalence). These factors may have acted as confounders and influenced clinical outcomes such as PN duration or weight gain. Due to limited availability of patient-level data and inconsistent reporting across studies, subgroup analyses or meta-regression could not be reliably performed. These differences likely reflect selection bias and may have influenced the observed outcomes.

The reliance on case series data further introduces the risk of spurious associations, as many included studies lacked control groups or adequate adjustment for confounders. The small number of included studies and patients limits the generalizability of our findings. Additional potential biases include the conversion of medians to means and variability in stoma location, underlying disease, and timing of intervention. Comparing MFR techniques is challenging due to the diversity of outcome metrics, which makes standardization difficult. In light of this, a “before and during MFR” paired data analysis may better assess the impact of the intervention.

Publication bias remains a concern, as studies reporting favorable or statistically significant results are more likely to be published. Moreover, the quality of the included studies was variable, and most analyses relied on aggregate rather than individual patient data, which restricted the ability to conduct more refined analyses or adjust for confounding variables. Clinical decision-making biases may also be present; for example, clinicians aware of MFR initiation might advance feeds earlier, introducing performance bias.

Nevertheless, the results for specific outcomes warrant careful interpretation. For cholestasis, low heterogeneity (*I*^2^ = 0.00%) ensured consistent findings across models, showing a clear reduction in risk. However, bilirubin levels presented a more complex picture. Moderate-to-high heterogeneity (*I*^2^ = 67.12% and *I*^2^ = 96.27% for peak bilirubin) led to divergent results between fixed- and random-effects models. While fixed-effects models suggested stronger effects, they may overstate the findings given the underlying variability. For PN duration, both models indicated significant reductions, but the random-effects model provided a more conservative estimate, reflecting inter-study variability. Despite the observed benefits, these findings must be interpreted with caution.

## 5. Conclusions

In conclusion, MFR may represent a promising strategy to support growth and reduce PN-related complications in newborns with enterostomy. While intestinal absorption was not directly measured in the included studies, the observed reduction in cholestasis and parenteral nutrition duration may suggest improved intestinal function. These findings highlight the potential benefits of MFR, although current evidence remains limited by methodological heterogeneity and study design.

Despite encouraging outcomes, challenges remain in standardizing protocols and minimizing complications. Further research should explore alternative techniques, refine safety assessments, and address concerns such as bacterial translocation. Prospective studies with systematic monitoring, including blood cultures, will be essential to confirm the safety of this approach.

While MFR shows promise, we emphasize the need for scientific rigor before recommending widespread implementation. Robust, well-designed studies demonstrating efficacy and safety are needed to support its potential integration into standard clinical practice for neonatal intestinal failure.

## Figures and Tables

**Table 1:** Summary of complications. Central Venous Catheter (CVC). Necrotizing Enterocolitis (NEC). Mucous Fistula Refeeding (MFR). Multi-Organ Failure (MOF).

Complication	Reported in
Dislodgement	Haddock et al. [19], Lau et al. [21], Gause et al. [28] (unprecised number)
Backflow	Pratap et al. [26], Haddock et al. [19], Lau et al. [21]
Wound infection	2 patients in Elliott et al. [23]
Peristomal dermatitis	3 patients in Elliott et al. [23] and in Lee et al. [33]
Entero-cutaneous fistula	1 patient in Elliott et al. [23]
Sepsis non-CVC related	3 patients in Wong et al. [1], 9 patients in Haddock et al. [19], and 5 patients in Lee et al. [33]
Diarrhea	Pratap et al. [26]
Fistula Prolapse	1 patient in Elliott et al. [23], 2 patients in Bindi et al. [30], and in Lee et al. [33]
Fistula Stenosis	1 patient in Bindi et al. [30]
Perforation	3 patients in Haddock et al. [19], and 1 patient in Lee et al. [33]
Hemorrhage	1 patient in Haddock et al. [19], and 2 patients in Bindi et al. [30]
Bowel distention	Pratap et al. [26] and in Lee et al. [33]
General mortality (n°)	4 patients with NEC in Wong et al. [1], 4 patients in Haddock et al. [19], 1 patient in Zornoza-Moreno et al. [29], 1 patient in Bindi et al. [30]
General mortality (details)	In Wong et al. [1]: 3 patients died from respiratory sepsis and 1 during cardiac surgery; In Haddock et al. [19]: 1 patient died from MOF after stoma closure, 1 from respiratory failure, 1 from septic cardiorespiratory failure, 1 from MFR-related MOF; In Zornoza-Moreno et al. [29]: 1 patient died from prematurity complications; In Bindi et al. [30]: 1 patient with NEC died after closure of the stoma

## Supplementary Materials

The following supporting information can be downloaded at: https://www.mdpi.com/article/10.3390/nu17152490/s1, Figure S1. Disease distribution by Mucous Fistula Refeeding (MFR); Figure S2. Stoma location by Mucous Fistula Refeeding (MFR); Table S1. Search strategies; Table S2. Methodological Quality and Risk of Bias Assessment of Included Studies Using the JBI Critical Appraisal Checklist; Table S3. Characteristics of included studies; Table S4. Mucous Fistula Refeeding (MFR) methods; Table S5. Funnel plots; Table S6. Overview of Results.

## Abbreviations

The following abbreviations are used in this manuscript:

MF	Mucous Fistula
PN	Parenteral Nutrition
BCR	Bowel Continuity Restoration
ESPEN	European Society for Clinical Nutrition and Metabolism
MFR	Mucous Fistula Refeeding
PRISMA	Preferred Reporting Items for Systematic Reviews and Meta-Analyses
NICU	Neonatal Intensive Care Unit
JBI	Joanna Briggs Institute
RoB	Risk of Bias
SD	Standard Deviation
IQR	Interquartile Range
ORs	Odds Ratios
CI	Confidence Intervals
RCT	Randomized Controlled Trial
CEST	Continuous Extracorporeal Stool Transport
