# Supplementary material for: Mucous Fistula Refeeding in Newborns: Why, When, How, and Where? Insights from a Systematic Review
Source: Nutrients. 2025 Jul 30;17(15):2490. doi: 10.3390/nu17152490 (PMC12348941; doi:10.3390/nu17152490)
Supplement: Supplementary file 1 [file nutrients-17-02490-s001.zip › Supplementary Material – Figure S1.pdf]

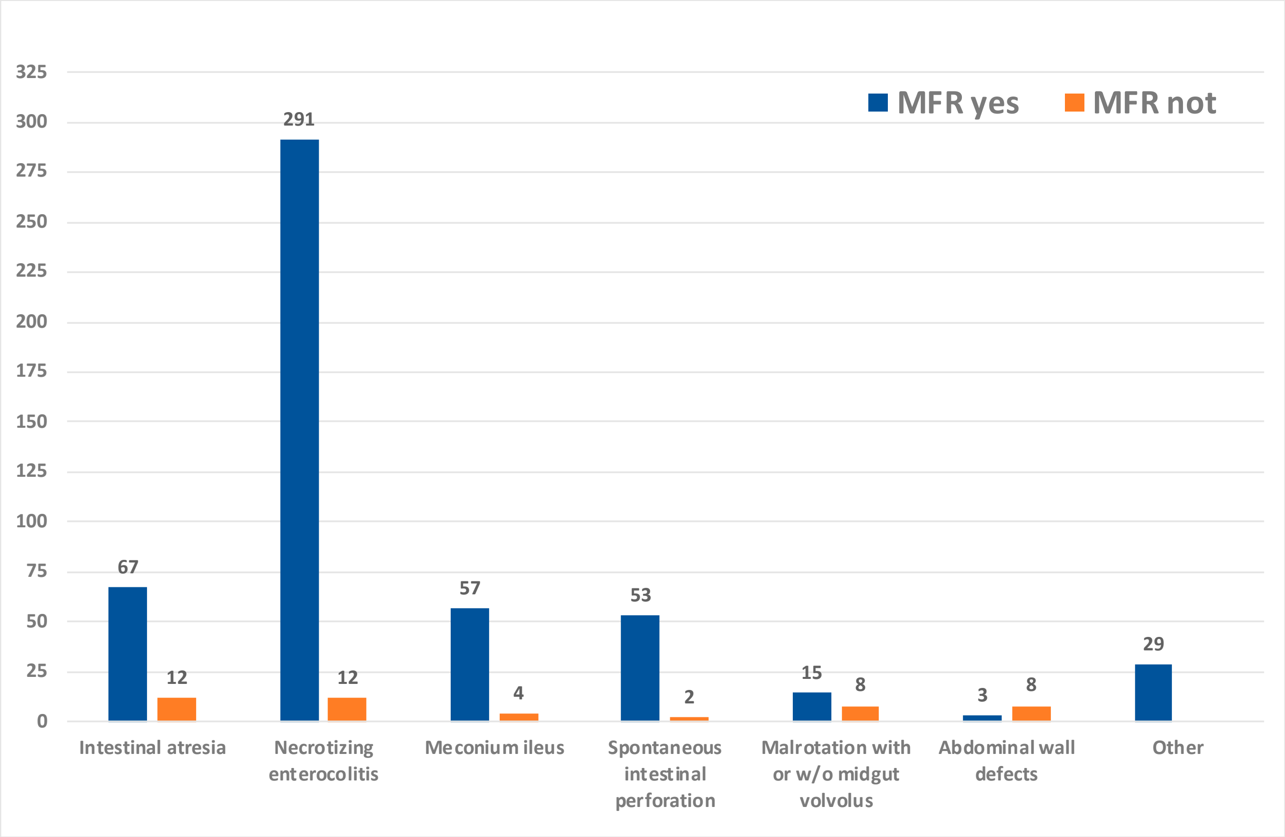

Supplementary Material – Figure S1. Disease distribution by Mucous Fistula Refeeding (MFR). Bar chart showing disease distribution among newborns with temporary double enterostomy receiving MFR (A) and not receiving MFR (B) in the current literature. MFR, mucous fistula refeeding.
