# Supplementary material for: Mucous Fistula Refeeding in Newborns: Why, When, How, and Where? Insights from a Systematic Review
Source: Nutrients. 2025 Jul 30;17(15):2490. doi: 10.3390/nu17152490 (PMC12348941; doi:10.3390/nu17152490)
Supplement: Supplementary file 1 [file nutrients-17-02490-s001.zip › Supplementary Material – Figure S2.pdf]

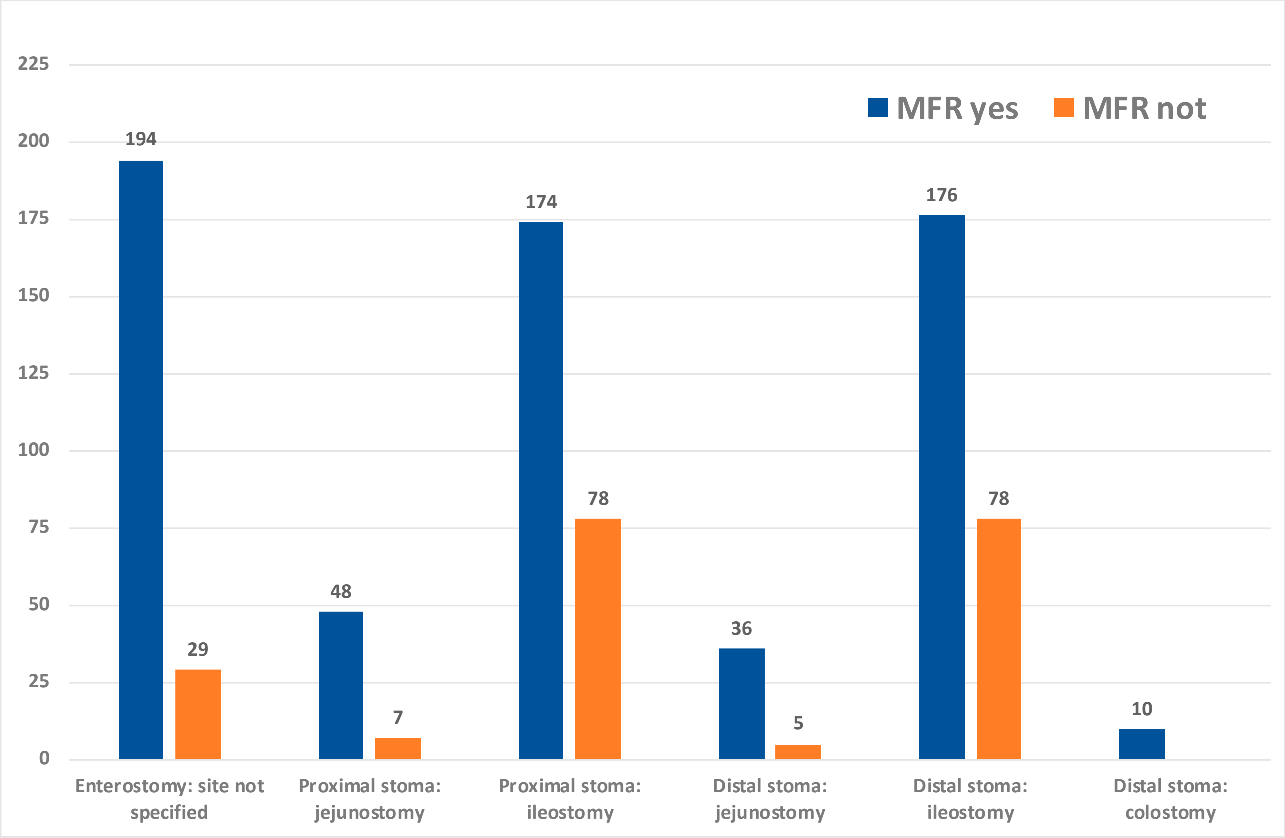

Supplementary Material – Figure S2. Stoma location by Mucous Fistula Refeeding (MFR). Bar chart showing stoma location in newborns with temporary double enterostomy receiving MFR (A) and controls (B) in the current literature. MFR, mucous fistula refeeding.
