# Supplementary material for: Mucous Fistula Refeeding in Newborns: Why, When, How, and Where? Insights from a Systematic Review
Source: Nutrients. 2025 Jul 30;17(15):2490. doi: 10.3390/nu17152490 (PMC12348941; doi:10.3390/nu17152490)
Supplement: Supplementary file 1 [file nutrients-17-02490-s001.zip › Supplementary Material – Table S1.pdf]

Database	Search strategy
**PubMed** ***database** <br>N°79	((((((ostomy) OR (ileostomy)) OR (jejunostomy)) OR (colostomy)) OR (mucous fistula)) OR (distal mucous fistula)) AND (recycling OR (refeeding)) <br>Search: **((((((ostomy) OR (ileostomy)) OR (jejunostomy)) OR (colostomy)) OR (mucous fistula)) OR (distal mucous fistula)) AND (recycling OR (refeeding)) - Saved search** Filters: **Child: birth-18 years, Newborn: birth-1 month, Infant: birth-23 months, Infant: 1-23 months** Sort by: **Most Recent** (("ostomy"[MeSH Terms] OR "ostomy"[All Fields] OR "ostomies"[All Fields] OR ("ileostomy"[MeSH Terms] OR "ileostomy"[All Fields] OR "ileostomies"[All Fields]) OR ("jejunostomy"[MeSH Terms] OR "jejunostomy"[All Fields] OR "jejunostomies"[All Fields]) OR ("colostomy"[MeSH Terms] OR "colostomy"[All Fields] OR "colostomies"[All Fields]) OR (("mucus"[MeSH Terms] OR "mucus"[All Fields] OR "mucous"[All Fields]) AND ("fistula"[MeSH Terms] OR "fistula"[All Fields] OR "fistulas"[All Fields] OR "fistula s"[All Fields] OR "fistulae"[All Fields] OR "fistulaes"[All Fields])) OR (("distal"[All Fields] OR "distalization"[All Fields] OR "distalize"[All Fields] OR "distalized"[All Fields] OR "distalizer"[All Fields] OR "distalizers"[All Fields] OR "distalizes"[All Fields] OR "distalizing"[All Fields] OR "distally"[All Fields] OR "distals"[All Fields]) AND ("mucus"[MeSH Terms] OR "mucus"[All Fields] OR "mucous"[All Fields]) AND ("fistula"[MeSH Terms] OR "fistula"[All Fields] OR "fistulas"[All Fields] OR "fistula s"[All Fields] OR "fistulae"[All Fields] OR "fistulaes"[All Fields]))) AND ("recyclability"[All Fields] OR "recyclable"[All Fields] OR "recyclables"[All Fields] OR "recyclate"[All Fields] OR "recyclates"[All Fields] OR "recycler"[All Fields] OR "recyclers"[All Fields] OR "recycles"[All Fields] OR "recycling"[MeSH Terms] OR "recycling"[All Fields] OR "recycle"[All Fields] OR "recycled"[All Fields] OR "recyclings"[All Fields] OR ("refeeding"[All Fields] OR "refeedings"[All Fields]))) AND (allchild[Filter] OR newborn[Filter] OR allinfant[Filter] OR infant[Filter]) **Translations** **ostomy:** "ostomy"[MeSH Terms] OR "ostomy"[All Fields] OR "ostomies"[All Fields] **ileostomy:** "ileostomy"[MeSH Terms] OR "ileostomy"[All Fields] OR "ileostomies"[All Fields] **jejunostomy:** "jejunostomy"[MeSH Terms] OR "jejunostomy"[All Fields] OR "jejunostomies"[All Fields] **colostomy:** "colostomy"[MeSH Terms] OR "colostomy"[All Fields] OR "colostomies"[All Fields] **mucous:** "mucus"[MeSH Terms] OR "mucus"[All Fields] OR "mucous"[All Fields] **fistula:** "fistula"[MeSH Terms] OR "fistula"[All Fields] OR "fistulas"[All Fields] OR "fistula's"[All Fields] OR "fistulae"[All Fields] OR "fistulaes"[All Fields] **distal:** "distal"[All Fields] OR "distalization"[All Fields] OR "distalize"[All Fields] OR "distalized"[All Fields] OR "distalizer"[All Fields] OR "distalizers"[All Fields] OR "distalizes"[All Fields] OR "distalizing"[All Fields] OR "distally"[All Fields] OR "distals"[All Fields] **mucous:** "mucus"[MeSH Terms] OR "mucus"[All Fields] OR "mucous"[All Fields] **fistula:** "fistula"[MeSH Terms] OR "fistula"[All Fields] OR "fistulas"[All Fields] OR "fistula's"[All Fields] OR "fistulae"[All Fields] OR "fistulaes"[All Fields] **recycling:** "recyclability"[All Fields] OR "recyclable"[All Fields] OR "recyclables"[All Fields] OR "recyclate"[All Fields] OR "recyclates"[All Fields] OR "recycler"[All Fields] OR "recyclers"[All Fields] OR "recycles"[All Fields] OR "recycling"[MeSH Terms] OR "recycling"[All Fields] OR "recycle"[All Fields] OR "recycled"[All Fields] OR "recyclings"[All Fields] **refeeding:** "refeeding"[All Fields] OR "refeedings"[All Fields]
**EMBASE** ***database** <br>N°188	('ostomy'/exp OR ostomy OR 'ileostomy'/exp OR ileostomy OR 'jejunostomy'/exp OR jejunostomy OR 'colostomy'/exp OR colostomy OR 'mucous fistula' OR (mucous AND ('fistula'/exp OR fistula)) OR 'distal mucous fistula' OR (distal AND mucous AND ('fistula'/exp OR fistula))) AND ('recycling'/exp OR recycling OR 'refeeding'/exp OR refeeding)
**CINAHL** ***database**	**Search Terms:** mucous fistula OR (ostomy or colostomy or ileostomy or stoma or ostomates or intestinal stoma) OR jejunostomy AND refeeding OR recycling

N°567	**Search Options**:<br>**Limiters** - Published Date: 19730101-20231231<br>**Expanders** - Apply equivalent subjects<br>**Narrow by Language:** - english<br>**Narrow by SubjectAge:** - infant: 1-23 months<br>**Narrow by SubjectAge:** - infant, newborn: birth-1 month<br>**Narrow by SubjectAge:** - all infant<br>**Search modes** - Boolean/Phrase<br><br>**Interface**<br>- EBSCOhost Research Databases<br><br>**Search Screen**<br>- Advanced Search<br><br>**Database**<br>- CINAHL Complete
**SCOPUS**<br>***database**<br><br>N°119	ALL (((((((ostomy) OR (ileostomy)) OR (jejunostomy)) OR (colostomy)) OR (mucous AND fistula)) OR (distal AND mucous AND fistula)) AND (refeeding OR (recycling))) AND (LIMIT-TO (LANGUAGE , "English") OR LIMIT-TO (LANGUAGE , "French")) AND (LIMIT-TO (EXACTKEYWORD , "Newborn") OR LIMIT-TO (EXACTKEYWORD , "Infant, Newborn") OR LIMIT-TO (EXACTKEYWORD , "Infant") OR LIMIT-TO (EXACTKEYWORD , "Child"))
**UpToDate**<br>***register**<br><br>N°3	Ostomy refeeding
**Cochrane central register of controlled study**<br>***register**<br><br>N°3	Mucous fistula refeeding
**Web of Science**<br>***database**<br><br>N°22	((((((ostomy) OR (ileostomy)) OR (jejunostomy)) OR (colostomy)) OR (mucous fistula)) OR (distal mucous fistula)) AND (recycling OR (refeeding))<br>Web of Science Categories: Pediatrics

Table S1. Search strategies
