# Supplementary material for: Mucous Fistula Refeeding in Newborns: Why, When, How, and Where? Insights from a Systematic Review
Source: Nutrients. 2025 Jul 30;17(15):2490. doi: 10.3390/nu17152490 (PMC12348941; doi:10.3390/nu17152490)
Supplement: Supplementary file 1 [file nutrients-17-02490-s001.zip › Supplementary Material – Table S2.pdf]

**Cohort**	**Schäfer et al (2000)**	**Drenckpoh et al (2012)**	**Koike et al (2015)**	**Lau et al (2016)**	**Gause et al (2016)**	**Yabe et al (2019)**	**Bindi et al (2020)**	**Woods et al (2021)**	**Coles et al (2022)**
**Q1**: Were the two groups similar and recruited from the same population?	🟢	🟢	🟢	🟢	🟢	🟢	🟢	🟢	🟢
**Q2**: Were the exposures measured similarly to assign people to both exposed and unexposed groups?	🟢	🟢	🟢	🟢	🟢	🟢	🟢	🟢	🟢
**Q3**: Was the exposure measured in a valid and reliable way?	🟢	🔴	🟢	🟢	🟢	🟢	🟢	🟢	🟢
**Q4**: Were confounding factors identified?	🟢	🟢	🟢	🔴	🔴	🔴	🔴	🟢	🔴
**Q5**: Were strategies to deal with confounding factors stated?	🟢	🟢	🟢	🟡	🟡	🟡	🟡	🟢	🟡
**Q6**: Were the groups/participants free of the outcome at the start of the study (or at the moment of exposure)?	🔵	🔴	🟢	🟡	🟡	🟡	🟡	🟡	🟡
**Q7**: Were the outcomes measured in a valid and reliable way?	🟢	🟢	🟢	🟢	🟢	🟢	🔴	🔴	🟢
**Q8**: Was the the follow up time reported and sufficient to be long enough for outcomes to occur?	🟢	🟢	🟢	🟢	🟢	🟢	🟢	🟢	🟢
**Q9**: Was follow up complete, and if not, were the reasons to loss to follow up described and explored?	🟢	🔴	🔴	🔴	🔴	🔴	🔴	🔴	🔴
**Q10**:Were strategies to address incomplete follow up utilized?	🔴	🔴	🔴	🔴	🔴	🔴	🔴	🔴	🔴
**Q11**: Was appropriate statistical analysis used?	🟡	🟢	🟢	🟢	🟢	🟢	🟢	🟢	🟢
**Column risk (%)**	8/11 (73%)	7/11 (64%)	9/11 (82%)	6/11 (55%)	6/11 (55%)	6/11 (55%)	5/11 (45%)	7/11 (64%)	6/11 (55%)
**Overall Risk of Bias**	Low	Moderate	Low	Moderate	Moderate	Moderate	High	Moderate	Moderate

**Case series**	**Al-Harbi et al (1999)**	**Wong et al (2004)**	**Pratap et al (2007)**	**Haddock et al (2015)**	**Zornoza-Moreno et al (2018)**	**Elliott et al (2019)**	**Sancar et al (2020)**
**Q1**: Were there clear criteria for inclusion in the case series?	🟢	🟢	🟢	🟢	🟢	🟢	🟢
**Q2**: Was the condition measured in a standard, reliable way for all participants included in the case series?	🟢	🟢	🟢	🟢	🟢	🟢	🟢
**Q3**: Were valid methods used for identification of the condition for all participants included in the case series?	🟢	🟢	🟢	🟢	🟢	🟢	🟢
**Q4**: Did the case series have consecutive inclusion of participants?	🟢	🟢	🟢	🟢	🟢	🟢	🟢
**Q5**: Did the case series have complete inclusion of participants?	🔵	🔵	🔵	🔵	🔵	🟢	🔵
**Q6**: Was there clear reporting of the demographics of the participants in the study?	🟢	🟢	🟢	🟢	🟢	🟢	🟢
**Q7**: Was there clear reporting of clinical information of the participants?	🟢	🟢	🟢	🟢	🟢	🟢	🟢
**Q8**: Were the outcomes or follow up results of cases clearly reported?	🟢	🟢	🟢	🟢	🟢	🟢	🟢
**Q9**: Was there clear reporting of the presenting site(s)/clinic(s) demographic information?	🟢	🟢	🟢	🟢	🟢	🟢	🟢
**Q10**: Was statistical analysis appropriate?	🟡	🟢	🟢	🟡	🟡	🟢	🟡
**Column risk (%)**	8/10 (80%)	9/10 (90%)	9/10 (90%)	8/10 (80%)	8/10 (80%)	10/10 (100%)	8/10 (80%)
**Overall Risk of Bias**	Low	Low	Low	Low	Low	Low	Low

**Randomized Controlled Trial**	**Lee et al (2023)**
**Q1**: Was true randomization used for assignment of participants to treatment groups?	🟢
**Q2**: Was allocation to treatment groups concealed?	🟢
**Q3**: Were treatment groups similar at the baseline?	🟢
**Q4**: Were participants blind to treatment assignment?	🟢
**Q5**: Were those delivering the treatment blind to treatment assignment?	🟢
**Q6**: Were treatment groups treated identically other than the intervention of interest?	🟢
**Q7**: Were outcome assessors blind to treatment assignment?	🔴
**Q8**: Were outcomes measured in the same way for treatment groups?	🟢
**Q9**: Were outcomes measured in a reliable way	🟢
**Q10**: Was follow up complete and if not, were differences between groups in terms of their follow up adequately described and analysed?	🟢
**Q11**: Were participants analysed in the groups to which they were randomized?	🟢
**Q12**: Was appropriate statistical analysis used?	🟢
**Q13**: Was the trial design appropriate and any deviations from the standard RCT design (individual randomization, parallel groups) accounted for in the conduct and analysis of the trial?	🟢
**Column risk (%)**	12/13 (92%)
**Overall Risk of Bias**	Low

**Judgement**	
Yes	🟢
No	🔴
Unclear	🔵
Not applicable	🟡

Table S2. Methodological Quality and Risk of Bias Assessment of Included Studies Using the JBI Critical Appraisal Checklist (https://jbi.global/critical-appraisal-tools). Quality assessment of the included studies using the Joanna Briggs Institute (JBI) Critical Appraisal Checklist for Case series, Cohort, Randomized Controlled Trials, with corresponding overall Risk of Bias scores. This figure provides an overview of the methodological rigor and risk of bias across the studies included in the review.
