# Supplementary material for: Mucous Fistula Refeeding in Newborns: Why, When, How, and Where? Insights from a Systematic Review
Source: Nutrients. 2025 Jul 30;17(15):2490. doi: 10.3390/nu17152490 (PMC12348941; doi:10.3390/nu17152490)
Supplement: Supplementary file 1 [file nutrients-17-02490-s001.zip › Supplementary Material – Table S3.pdf]

Study	Publication year	Country	Database source	Type of study	Design of the study	Patients included (n°)	Patients with MFR (n°)	Male (%)
Al-Harbi et al.	1999	Canada	Single center	Case series	Retrospective	6	6	66.7
Schäfer et al.	2000	Germany	Single center	Cohort	Ambispective	59	59	-
Wong et al.	2004	China	Single center	Case series	Retrospective	12	12	41.7
Pratap et al.	2007	Nepal	Single center	Case series	Retrospective	10	10	80.0
Drenckpoh et al.	2012	United States of America	Single center	Cohort	Retrospective	29	29	51.7
Haddock et al.	2015	Canada	Single center	Case series	Retrospective	23	23	43.5
Koike et al.	2016	Japan	Single center	Cohort	Retrospective	27	13	63.0
Lau et al.	2016	China	Single center	Cohort	Retrospective	92	77	58.7
Gause et a.	2016	United States of America	Single center	Cohort	Retrospective	24	13	45.8
Zornoza-Moreno et al.	2018	Mexico	Single center	Case series	Retrospective	7	7	-
Yabe et al.	2019	Japan	Single center	Cohort	Retrospective	16	10	62.5
Elliott et al.	2019	Canada	Canadian Neonatal Network	Case series	Retrospective	31	31	45.2
Bindi et al.	2020	Italy	Single center	Cohort	Retrospective	85	65	65.9
Sancar et al.	2020	Austria	Single center	Case series	Retrospective	23	23	39.1
Woods et al.	2021	United States of America	Single center	Cohort	Retrospective	101	65	57.4
Coles et al.	2022	United Kingdom	Single center	Cohort	Retrospective	71	29	62.0
Lee et al.	2023	Korea	Single center	Randomized controlled trial	Prospective	15	10	53.3

Table 1

Study	Gestation age at birth (weeks)	Weight at birth (g)	Age at first surgery (d)	Time at stoma closure (d)	Weight at stoma closure (g)	Total remaining bowel (cm)	Resected bowel (cm)	Daily weight gain BEFORE refeeding (g/d)	Daily weight gain DURING refeeding (g/d)	NICU stay (d)	Total hospital stay (d)	PN total lenght (d)
Al-Harbi et al.	31.7 (27-38) †	1530, 2450, 2460, 990, 533, 3400	13 (2-27) †	330; 90; 60; 90; 120; 70	2400 in one patient	36 in one patient	10 and 25 in two patients	-9.2 ± 21.2 *	28.4 ± 12.1 *			
Schäfer et al.	32.7 in MFR group and 35 in control group ■	1870 in MFR group and 2490 in control group ■	5 in MFR group and 7 in control group ■	106 in MFR group and 146 in control group ■	4060 in MFR group and 4650 in control group ■							
Wong et al.	31 (24-37) §	1590 ± 260 *				66.5 ± 12.6 *		10.5 ± 1.5 *	18.9 ± 2.9 *			
Pratap et al.	34.2 ± 4.6*	2580 ± 993 *		92 ± 4.2 *		35.5 ± 3.5 *		10.6 ± 6.2 *	17.7 ± 2.9 *		59.5 ± 11.9 *	
Drenckpoh et al.	30.3 ± 6 (23-41) * †	1635 ± 1146 (495-3970) * †										
Haddock et al.	35 (23-40)†	2416 (585-4294)†								117 ■	159 ■	133 (18-319) †
Koike et al.	34.4 (27-41) in MFR group and 33 (25-40) in control group †	2000 (570–3280) in MFR group and 2390 (720-3600) in control group †			2680 in MFR group ■	33.6 ± 14.6 in MFR group and 31.4 ± 21.2 in control group *						
Lau et al.	30.79 ± 0.51 (23-41) in MFR group and 30.53 ± 1.1 (24-38) in control group * †	1580 ± 90 (570-3780) in MFR group and 1530 ± 790 (710-2950) in control group * †				67.79 ± 4.23 in MFR group and 78.67 ± 10.14 in control group *				179.30 ± 33.09 (22–748) in MFR group and 262.90 ± 95.00 (11–423) in control group * †	125.70 ± 20.18 (10-268) in MFR group and 200.20 ± 63.28 (11-393) in control group * †	47.68 ± 5.84 (7-236) in MFR group and 135.80 ± 58.40 (4-360) in control group * †
Gause et a.	27.3 (24.9, 28.9) in MFR group and 27.4 (25.7, 28.4) in control group ‡	890 (690, 1680) in MFR group, 1000 (720, 1150) in control group ‡		52 (46, 80) in MFR group, 55 (42, 76) in control group ‡								30.5 (23.5, 465) in MFR group, 48 (41, 99) in control group ‡
Zornoza-Moreno et al.	35 (28-39) §	2000 (1900-3500) §	3 (1-180) §									
Yabe et al.	28 (23-33) in MFR group and 27 (23-37) in control group §	925 (502–1206) in MFR group and 976 (560–2366) in control group §	5 (1-15) in MFR group and 9 (1-22) in control group §	84 (68-216) in MFR group and 155 (76-254) in control group §	2100 (1580–4842) in MFR group and 2645 (2246–3082) in control group §							25 (5-88) in MFR group and 87 (63-162) in control group §
Elliott et al.	32 ± 4.5 *	2279 ± 1055 *		80 ± 34.5 *		70.5 ± 23.3 in twentytwo patients *	17.2 (2-45) in eleven patients †	1.5 ± 23.0 *	27.3 ±17.9 *	116 ± 53 *	145 ± 75.7 *	55 ± 31.4 *
Bindi et al.	28 (22-39) in MFR group and 26 (27-29) in control group †	1153.6 (430-4450) in MFR group and 1160 (510-1220) in control group †			2150 (1100-6020) in MFR group and 1528 (1070-3700) in control group †							

Sancar et al.	31.60 (24-38) †	1876.95 (640–4000) †		77 (45-179) †	2000 ■				
Woods et al.	26.9 (25.0, 29.6) in NEC MFR group, 26.7 (25.3, 29.1) in NEC non-MFR group, 34.1 (28.5, 36.6) in SBA MFR group, 27.2 (25.7, 34.4) in SBA non-MFR group ‡	840 (670, 1171) in NEC MFR group, 808 (620, 1160) in NEC non-MFR group, 1960 (1375, 2566) in SBA MFR group, 1085 (760, 2390) in SBA non-MFR group ‡	29 (17,41) in NEC MFR group, 26 (12,45) in NEC non-MFR group, 2 (1,8) in SBA MFR group, 6 (2,12) in SBA non-MFR group ‡	50 (42, 62) in NEC MFR group, 50 (41, 61) in NEC non-MFR group, 51 (48, 66) in SBA MFR group, 54 (47, 66) in SBA non-MFR group ‡			12.0 (8.8, 22.0) in NEC MFR group, 8.4 (6.0, 12.0) in NEC non-MFR group, 5.5 (1.0, 11.6) in SBA MFR group, 5.0 (3.0, 8.7) in SBA non-MFR group ‡		
Coles et al.	26 (23-39) in MFR group and 27 (23-40) in control group §	835 (585–2895) in MFR group and 950 (500–3600) in control group §		54.5 (24–211) in MFR group and 56 (33-169) in control group §					
Lee et al.	25 (23-26+4) in High output MFR group and 26+6 (26+2-33+5) in normal output MFR group and 29 (27+4-30+2) in control group §	540 (520, 620) in MFR high-output enterostomy group, 880 (620, 1240) MFR low-output enterostomy group and 1.190 (850,1500)in control group §	12 (6-43) in high output MFR group, 13 (2-28) in normal output MFR group, and 5 (1-18) in control group §	103 (73-123) in MFR group and 85 (64-122) in control group §			10 (0-35) in MFR group and 4 (0-50) in control group §	9.91 ± 3.20 *	25.21 ± 5.34 *

Table 1 continued

Table S3. Characteristics of included studies. Mucous Fistula Refeeding (MFR). Neonatal Intensive Care (NICU). Parenteral Nutrition (PN). Legend: * mean ± SD; † mean (min-max); ‡ median (25th, 75th, %ile); § median (min-max); ■ mean; ▲ min - max
