# Supplementary material for: Mucous Fistula Refeeding in Newborns: Why, When, How, and Where? Insights from a Systematic Review
Source: Nutrients. 2025 Jul 30;17(15):2490. doi: 10.3390/nu17152490 (PMC12348941; doi:10.3390/nu17152490)
Supplement: Supplementary file 1 [file nutrients-17-02490-s001.zip › Supplementary Material – Table S4.pdf]

Study	Indication for MFR	Interval first surgery - start MFR (days)	MFR duration (days)	Contrast enema / flush pre-refeeding	Operator	Substance refed
Al-Harbi et al.	High output enterostomy, electrolyte disturbance, failure to achieve adequate weight gain, no stricture on contrast study	21.4 (13-30)†	109.16 (30-330) †	Lower gastrointestinal series	Hospital + parents	Proximal stoma effluent
Schäfer et al.	To prevent the development of a non-used distal bowel and a SBS	31 ■				Proximal stoma effluent
	For stimulation of the non-used bowel and maintenance of an open lumen	28 ■	58 ■			Isotonic electrolyte solution
Wong et al.	If no stricture seen	26 (11 - 140) §	63.5 ± 15.7 *	Loopogram		Proximal stoma effluent
Pratap et al.	Presence of enterostomy	With return of stoma function	20 - 156 ▲		Hospital + parents	Proximal stoma effluent mixed with a semielemental diet containing medium-chain triglycerides (Pregestimil, Mead Johnson, USA)
Drenckpoh et al.						Proximal stoma effluent
Haddock et al.	Surgical anatomy of the patient and observation of the ostomy losses		36 (10 - 79)†	Loopogram	Surgical team in the operating room or the enterostomal therapist or nurses at the bedside	Proximal stoma effluent
Koike et al.	Improve nutritional status before stoma closure	39.5 (8-97) †	26.2 ± 14.9 (8 - 59) *†	Tube inserted from a distal enterostomy under fluoroscopy to confirm the safety of using the distal small bowel and its patency (radiographic contrast medium)		Proximal stoma effluent
Lau et al.	If no stricture was identified	28.7 ± 8.7 (15 - 140) *†	76.5 ± 5.5 (13 - 236) *†	Loopogram		Proximal stoma effluent
Gause et a.	Based on surgeon preference	After proximal bowel function confirmed (enterostomy productive for 24 h)			Attending surgeon	Proximal stoma effluent
Zornoza-Moreno et al.		120 (60 - 180) §	100 (30 - 180) §	Loopogram	Hospital + parents	Proximal stoma effluent mixed with 50 ml of normal saline
Yabe et al.		Sufficient proximal stoma output obtained		Loopogram	NICU nurses	Proximal stoma effluent
Elliott et al.	Poor growth with need for TPN and a large portion of distal bowel that could be used	29 ± 25 *	41 ± 22 *	Lower gastrointestinal series		Proximal stoma effluent
Bindi et al.		10 - 15 ▲		Loopogram	Surgeon	5 ml 0.9% sodium chloride once a day for 3 days. Then, 20 ml of stoma's output twice a day +/- 0.9% sodium chloride (maximum 10 ml)
Sancar et al.	Premature and mature newborns with enterostomies, irrespective of bowel resection	5 ■	57 ■		Surgeon and nursing staff	Drip saline infusion
Woods et al.	Pediatric surgeon and neonatologist make the clinical decision	Enteral feeds started and clinical condition stabilized	25 (19, 29) in NEC MFR group, 31 (27, 39) in SIP n MFR group ‡	Loopogram		Proximal stoma effluent
Coles et al.	Reason for not recycling : Inability to catheterise the mucous fistula and distal disease (stricture/ongoing NEC/microcolon) "not an inpatient" and "patient too unwell"	31 (5 - 66) §	15.5 (1 - 51) §	Loopogram	Surgeon and nursing staff	Proximal stoma effluent. If small amount of stool it was mixed with saline, to ensure an adequate volume
Lee et al.	Enterostomy effluent volume ≥ 40 mL/kg/day			Loopogram	Initially performed by the surgeon and continued by the NICU nurse	Proximal stoma effluent

Collection details			Refeeding details									
**Study**	**Collection modalities**	**Interval of collection**	**In the distal stoma: tube type**	**In the distal stoma: tube size**	**Distance advanced**	**Secured modality**	**MF skin protection**	**Per rectum**	**Length of refeeding**	**Method**	**Rate**	**Times per day**
Al-Harbi et al.	Emptied into an appropriately sized Luer lock syringe and collected in a regular stoma collection bag	4-6 h	Feeding tube, Foley, red rubber, urinary catheter	8 Fr	5 cm	Extra thin DuoDerm	Coloplast:3210 skin barrier or Karaya powder		Intermittent	Syringe infusion pump connected to regular IV infusion tubing		
Schäfer et al.	Newly developed adhesive stoma bag connected to a silicon tube		Foley catheter	8-10 Fr		Balloon inflated with 0.5 to 1 ml for 10 h twice a day			20 h	Roller pump	1-15 mL/h	
									20 h			
Wong et al.		4-6 h	Nasogastric tube	8 Fr		Adhesive tape tunneled through a small hole in a stoma bag				Syringe pump		
Pratap et al.	8 Foley catheter inserted for 5 cm and clamped. Clamp released to collect the effluent in a Luer syringe	4-6 h	Foley catheter	8 Fr	5 cm			Foley clamped released every 6 h or if abdominal distension	Continuous	Microdrip infusion		
Drenckpoh et al.	Collected from the ostomy bag and stored in an enteral feeding syringe	8 h	Red rubber catheter tube through the port in the ostomy bag		5 cm	Tube gauze tape transpore			Over 4-8 h	Enteral pump		
Haddock et al.			Variety of feeding catheters			If dislodgement or back flow: advanced down the MF under fluoroscopic guidance by interventional radiologist			Continuous or intermittent		10.5 (range 0.5–23 mL/h)	
Koike et al.	Stool collected, filtrated by dry gauze and aspirated into a syringe	4 h	New Enteral Feeding Tube (Covidien, Japan)	5 or 6.5 Fr	3-8 cm	Attached by an ostomy pouch through a small hole with waterproof film			Within 4 h	Syringe pump	ns	
Lau et al.		4 h	Feeding tube	8 Fr		Adhesive tape			Continuous	Syringe pump		
Gause et a.	Succus entericus collected from the proximal enterostomy		Foley catheter	6 Fr		Balloon inflated with 0.5 ml of saline			Continuous	Electronic feeding pump		
Zornoza-Moreno et al.	Collection of the stool mixed with 50 ml of normal saline and filtered		Foley catheter	16 Fr		Balloon minimally inflated			Intermittent	Syringe 20 mL	3 mL/min	3
Yabe et al.	Collection of proximal stoma output pooled in the pouches	3 h	Catheter	7 Fr					Over 5-10 min	Manual syringe		
Elliott et al.	Stool collected to coincide with the feeding and handling times of the neonate. Proximal effluent aspirated into a lock syringe	3-4 h	Foley catheter	8 Fr		With or without a balloon inflated	Coloplast skin barrier (circular with a hole in the middle no larger than MF diameter), replacing it whenever it became soiled		Continuous	Syringe pump		
Bindi et al.	Manual aspiration		Nelaton catheter	6-8 Fr if > 2 kg or directly with syringe <2 kg	3-5 cm	Without fixation				Manual syringe 5 ml		2
Sancar et al.	Proximal stoma content accumulated in the ostomy tract		Feeding probe	5-8 Fr	3-5 cm			Special self-controlling catheter		Manual injection		
Woods et al.		4 h	Foley catheter or feeding tube	6 Fr / 5 or 6.5 Fr	4-5 cm	Tape or balloon filled with 0.5 to 1 mL sterile water or saline, released every 12 hours for 5 to 10 minutes to allow for mucosal rest			Continuous	Syringe pump	1 mL/h, increased stepwise to a rate, either equal to the hourly enterostomy output or 4-8 mL/h	2
Coles et al.			Soft rubber catheter removed at the end of every refeeding		3-10 cm				Intermittent	Manual, using gravity method		1 to 4
Lee et al.	Proximal stoma output collected in pouches every 8 h		Nelaton catheter, new catheter for each infusion and subsequently removed	3 or 4 Fr					Over 5-10 min	Manual		Initially, 3 times. If no side effects, 50% and 100% administered 6 times.

Table S4. Mucous Fistula Refeeding (MFR) methods. Legend: * mean ± SD; † mean (min-max); ‡ median (25th, 75th, %ile); § median (min-max); ■ mean;

▲ min - max
