# Supplementary material for: Mucous Fistula Refeeding in Newborns: Why, When, How, and Where? Insights from a Systematic Review
Source: Nutrients. 2025 Jul 30;17(15):2490. doi: 10.3390/nu17152490 (PMC12348941; doi:10.3390/nu17152490)
Supplement: Supplementary file 1 [file nutrients-17-02490-s001.zip › Supplementary Material – Table S5.pdf]

**MEAN WEIGHT GAIN**

AL-HARBI

```
. ttesti 6 -9.2 21.2 6 28.4 12.1, unequal

Two-sample t test with unequal variances
------------------------------------------------------------------------------
         |     Obs        Mean    Std. Err.   Std. Dev.   [95% Conf. Interval]
---------+--------------------------------------------------------------------
       x |       6        -9.2    8.654864        21.2   -31.44804    13.04804
       y |       6        28.4    4.939804        12.1    15.70183    41.09817
---------+--------------------------------------------------------------------
combined |      12         9.6    7.396011    25.62054   -6.678511    25.87851
---------+--------------------------------------------------------------------
    diff |               -37.6    9.965357               -60.60783   -14.59217
------------------------------------------------------------------------------
    diff = mean(x) - mean(y)                                      t =  -3.7731
Ho: diff = 0                     Satterthwaite's degrees of freedom =  7.94508
    Ha: diff < 0                 Ha: diff != 0                 Ha: diff > 0
 Pr(T < t) = 0.0028         Pr(|T| > |t|) = 0.0055          Pr(T > t) = 0.9972
```

WONG

```
. ttesti 12 -10.5 1.5 12 18.9 2.9, unequal

Two-sample t test with unequal variances
------------------------------------------------------------------------------
         |     Obs        Mean    Std. Err.   Std. Dev.   [95% Conf. Interval]
---------+--------------------------------------------------------------------
       x |      12       -10.5    .4330127         1.5   -11.45305   -9.546945
       y |      12        18.9    .8371579         2.9    17.05743    20.74257
---------+--------------------------------------------------------------------
combined |      24         4.2     3.09962    15.18498   -2.212053    10.61205
---------+--------------------------------------------------------------------
    diff |               -29.4    .9425144                -31.3932    -27.4068
------------------------------------------------------------------------------
    diff = mean(x) - mean(y)                                      t = -31.1932
Ho: diff = 0                     Satterthwaite's degrees of freedom =  16.4927
    Ha: diff < 0                 Ha: diff != 0                 Ha: diff > 0
 Pr(T < t) = 0.0000         Pr(|T| > |t|) = 0.0000          Pr(T > t) = 1.0000
```

YABE

```
. ttesti 10 -10.6 6.2 10 17.7 2.9, unequal

Two-sample t test with unequal variances
------------------------------------------------------------------------------
         |     Obs        Mean    Std. Err.   Std. Dev.   [95% Conf. Interval]
---------+--------------------------------------------------------------------
       x |      10       -10.6    1.960612         6.2   -15.03521   -6.164787
       y |      10        17.7    .9170605         2.9    15.62546    19.77454
---------+--------------------------------------------------------------------
combined |      20        3.55    3.412863    15.26279   -3.593204     10.6932
---------+--------------------------------------------------------------------
    diff |               -28.3    2.164486               -32.98511   -23.61489
------------------------------------------------------------------------------
    diff = mean(x) - mean(y)                                      t = -13.0747
Ho: diff = 0                     Satterthwaite's degrees of freedom =  12.7582
    Ha: diff < 0                 Ha: diff != 0                 Ha: diff > 0
 Pr(T < t) = 0.0000         Pr(|T| > |t|) = 0.0000          Pr(T > t) = 1.0000
```

ELLIOTT

```
. ttesti 31 1.5 23.0 31 27.3 17.9, unequal

Two-sample t test with unequal variances
------------------------------------------------------------------------------
         |     Obs        Mean    Std. Err.   Std. Dev.   [95% Conf. Interval]
---------+--------------------------------------------------------------------
       x |      31         1.5    4.130922          23   -6.936468    9.936468
       y |      31        27.3    3.214935        17.9    20.73423    33.86577
---------+--------------------------------------------------------------------
combined |      62        14.4    3.076657    24.22562    8.247845    20.55216
---------+--------------------------------------------------------------------
    diff |               -25.8    5.234532               -36.28363   -15.31637
------------------------------------------------------------------------------
    diff = mean(x) - mean(y)                                      t =  -4.9288
Ho: diff = 0                     Satterthwaite's degrees of freedom =  56.5875

    Ha: diff < 0                 Ha: diff != 0                 Ha: diff > 0
 Pr(T < t) = 0.0000         Pr(|T| > |t|) = 0.0000          Pr(T > t) = 1.0000
```

LEE

```
. ttesti 10 9.91 3.2 10 25.21 5.34, unequal

Two-sample t test with unequal variances
------------------------------------------------------------------------------
         |     Obs        Mean    Std. Err.   Std. Dev.   [95% Conf. Interval]
---------+--------------------------------------------------------------------
       x |      10        9.91    1.011929         3.2    7.620858    12.19914
       y |      10       25.21    1.688656        5.34    21.38999    29.03001
---------+--------------------------------------------------------------------
combined |      20       17.56    1.999507    8.942066    13.37498    21.74502
---------+--------------------------------------------------------------------
    diff |               -15.3    1.968644               -19.50289   -11.09711
------------------------------------------------------------------------------
    diff = mean(x) - mean(y)                                      t =  -7.7718
Ho: diff = 0                     Satterthwaite's degrees of freedom =  14.7255

    Ha: diff < 0                 Ha: diff != 0                 Ha: diff > 0
 Pr(T < t) = 0.0000         Pr(|T| > |t|) = 0.0000          Pr(T > t) = 1.0000
```

**CHOLESTASIS**

**RANDOM-EFFECTS MODEL**

```
. meta esize pnald_yes_inmfr pnald_no_inmfr pnald_yes_nonmfr pnald_no_nonmfr, studylabel(author)

Meta-analysis setting information

 Study information
    No. of studies: 3
       Study label: author
        Study size: _meta_studysize
      Summary data: pnald_yes_inmfr pnald_no_inmfr pnald_yes_nonmfr pnald_no_nonmfr

       Effect size
              Type: lnoratio
             Label: Log odds-ratio
          Variable: _meta_es
   Zero-cells adj.: None; no zero cells

         Precision
         Std. err.: _meta_se
                CI: [_meta_cil, _meta_ciu]
          CI level: 95%

  Model and method
             Model: Random effects
            Method: REML
```

**. meta summarize, random(reml) eform**

```
  Effect-size label: Log odds-ratio
        Effect size: _meta_es
          Std. err.: _meta_se
        Study label: author

Meta-analysis summary                     Number of studies =      3
Random-effects model                      Heterogeneity:
Method: REML                                          tau2 =  0.0000
                                                    I2 (%) =    0.00
                                                        H2 =    1.00

--------------------------------------------------------------------
            Study |     Odds ratio    [95% conf. interval]  % weight
------------------+-------------------------------------------------
              Lau |          0.259       0.076       0.885     37.07
             Yabe |          0.111       0.008       1.516      8.22
            Woods |          0.110       0.040       0.302     54.70
------------------+-------------------------------------------------
       exp(theta) |          0.151       0.071       0.319
--------------------------------------------------------------------
Test of theta = 0: z = -4.95                    Prob > |z| = 0.0000
Test of homogeneity: Q = chi2(2) = 1.17           Prob > Q = 0.5564
```

**. meta forestplot, random(reml)** esrefline nullrefline eform

```
  Effect-size label: Log odds-ratio
        Effect size: _meta_es
          Std. err.: _meta_se
        Study label: author
```

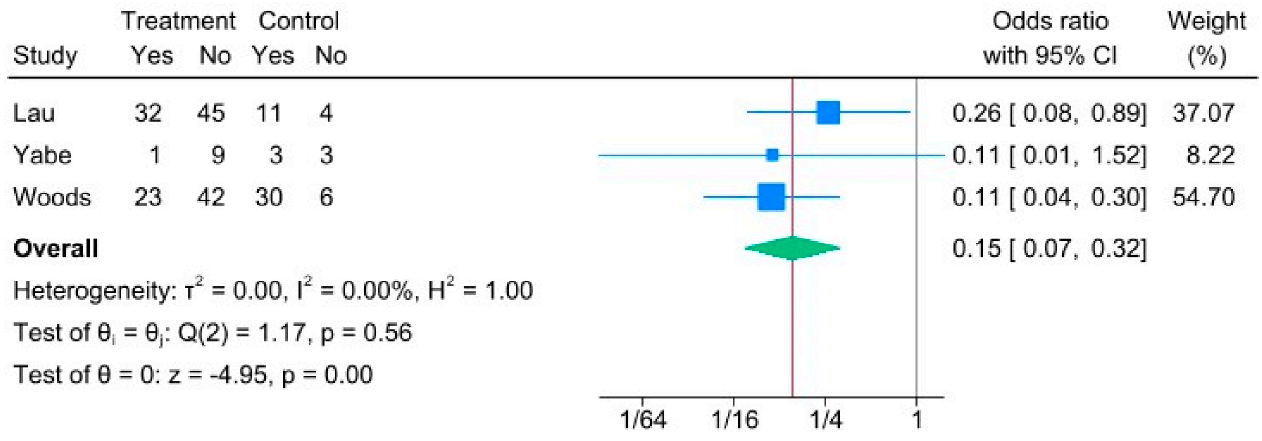


Random-effects REML model

```
. meta funnelplot

  Effect-size label: Log odds-ratio
        Effect size: _meta_es
          Std. err.: _meta_se
              Model: Common effect
             Method: Inverse-variance
```

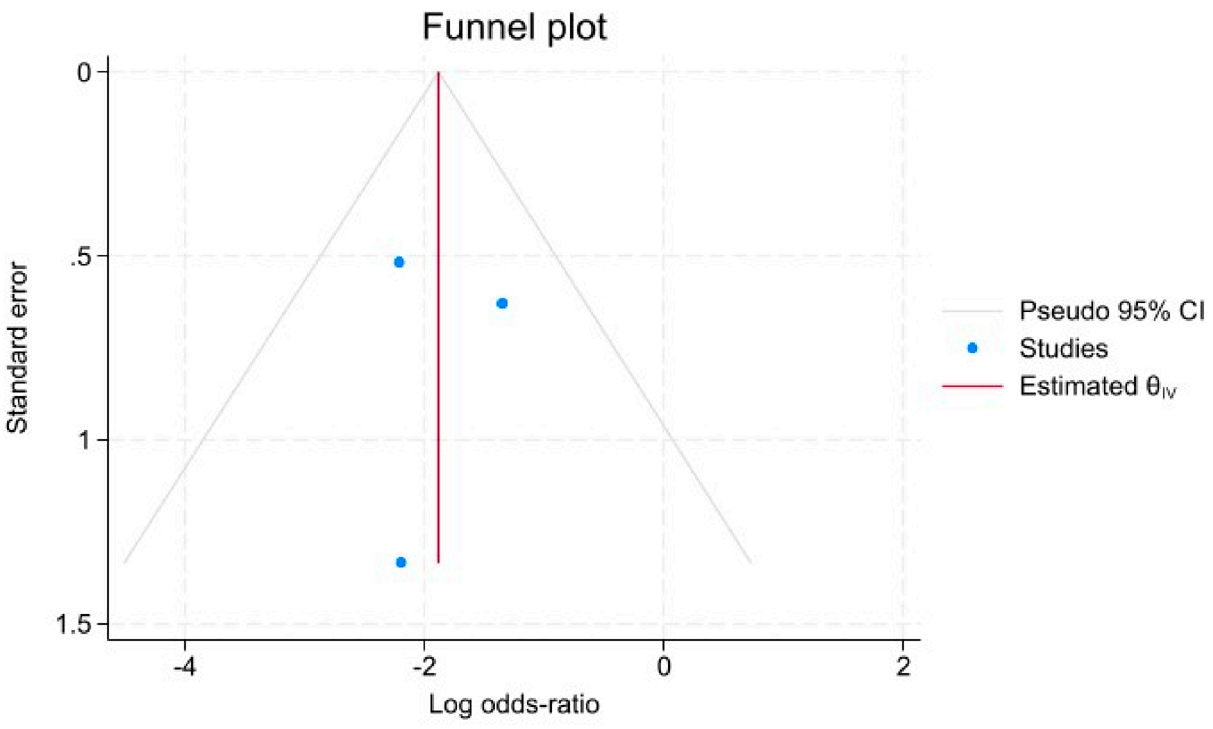


```
. meta labbeplot, random(reml)

  Effect-size label: Log odds-ratio
        Effect size: _meta_es
          Std. err.: _meta_se
       Summary data: pnald_yes_inmfr pnald_no_inmfr pnald_yes_nonmfr pnald_no_nonmfr
              Model: Random effects
             Method: REML
```

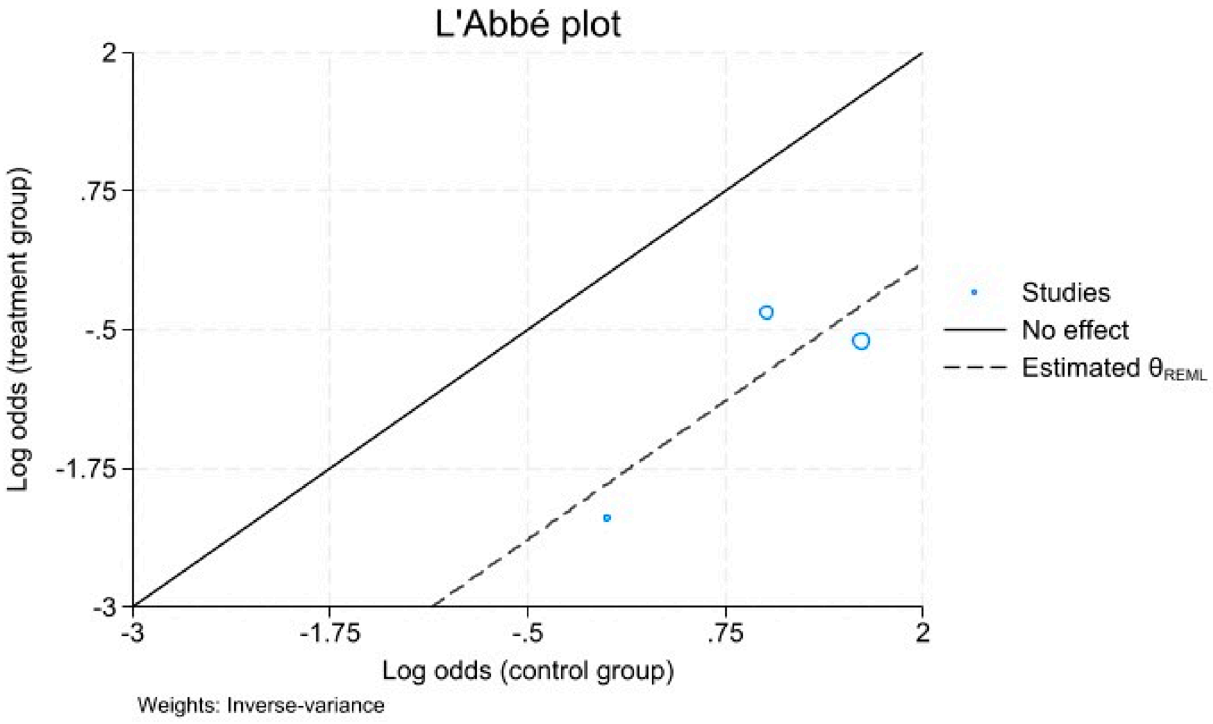


```
. meta galbraith, random(reml)

  Effect-size label: Log odds-ratio
        Effect size: _meta_es
          Std. err.: _meta_se
              Model: Random effects
             Method: REML
```

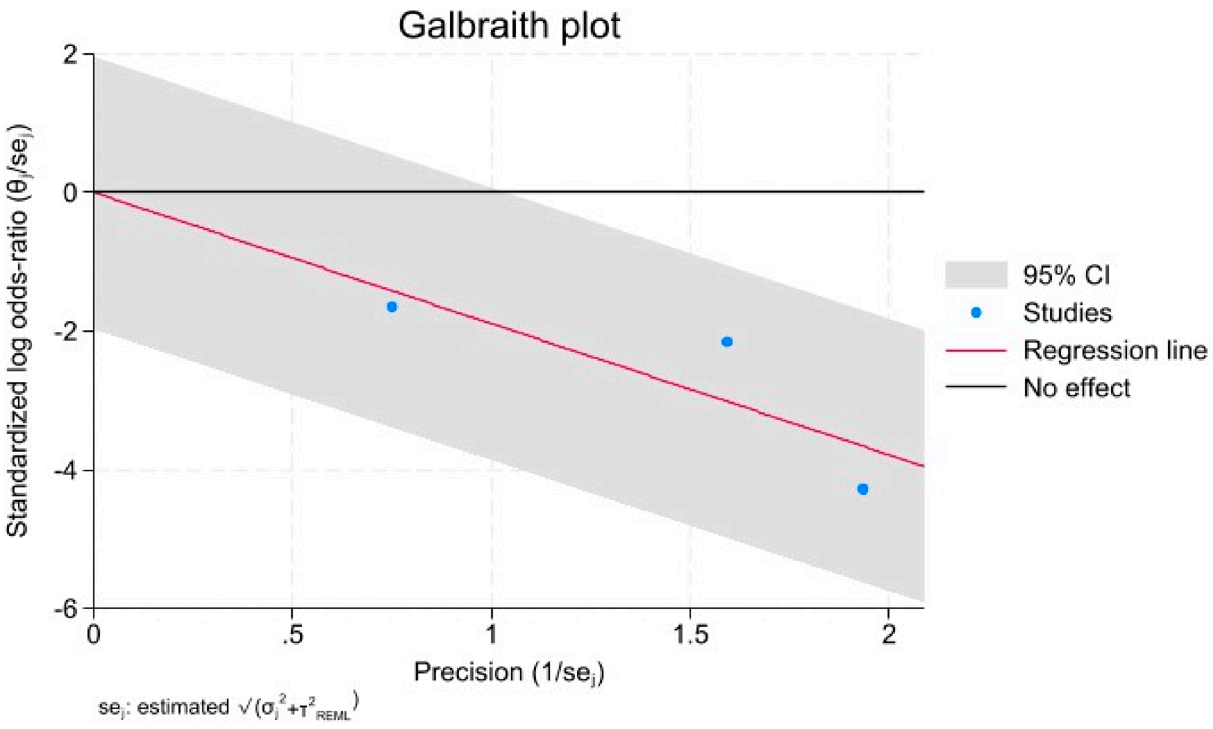


**FIXED-EFFECTS MODEL**

```
. meta summarize, fixed(ivariance) eform
```

```
  Effect-size label: Log odds-ratio
        Effect size: _meta_es
          Std. err.: _meta_se
        Study label: author

Meta-analysis summary                     Number of studies =      3
Fixed-effects model                       Heterogeneity:
Method: Inverse-variance                            I2 (%) =    0.00
                                                        H2 =    1.00

--------------------------------------------------------------------
            Study |     Odds ratio    [95% conf. interval]  % weight
------------------+-------------------------------------------------
              Lau |          0.259       0.076       0.885     37.07
             Yabe |          0.111       0.008       1.516      8.22
            Woods |          0.110       0.040       0.302     54.70
------------------+-------------------------------------------------
       exp(theta) |          0.151       0.071       0.319
--------------------------------------------------------------------
Test of theta = 0: z = -4.95                     Prob > |z| = 0.0000
Test of homogeneity: Q = chi2(2) = 1.17            Prob > Q = 0.5564
```

```
. meta forestplot, fixed(ivariance) esrefline nullrefline eform

  Effect-size label: Log odds-ratio
        Effect size: _meta_es
          Std. err.: _meta_se
        Study label: author
```

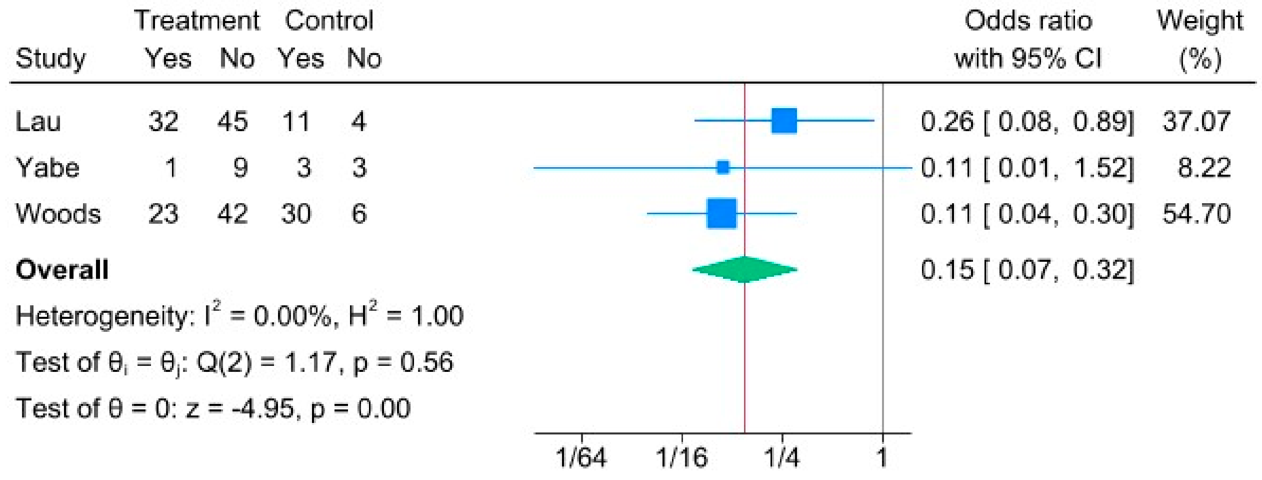


Fixed-effects inverse-variance model

```
. meta funnelplot

  Effect-size label: Log odds-ratio
        Effect size: _meta_es
          Std. err.: _meta_se
              Model: Common effect
             Method: Inverse-variance
```

*Graph overlapping with that obtained with the random-effects model*

```
. meta bias, egger

  Effect-size label: Log odds-ratio
        Effect size: _meta_es
          Std. err.: _meta_se

Regression-based Egger test for small-study effects
Random-effects model
Method: REML

H0: beta1 = 0; no small-study effects
            beta1 =     -0.03
      SE of beta1 =     1.803
                z =     -0.02
       Prob > |z| =    0.9865
```

```
. meta labbeplot, fixed(ivariance)

  Effect-size label: Log odds-ratio
        Effect size: _meta_es
          Std. err.: _meta_se
       Summary data: pnald_yes_inmfr pnald_no_inmfr pnald_yes_nonmfr pnald_no_nonmfr
              Model: Fixed effects
             Method: Inverse-variance
```

*Graph overlapping with that obtained with the random-effects model*

**CATHETER-RELATED SEPSIS**

**RANDOM-EFFECTS MODEL**

```
. meta esize sepsis_yes_inmfr sepsis_no_inmfr sepsis_yes_nonmfr sepsis_no_nonmfr,
studylabel(author)

Meta-analysis setting information

 Study information
    No. of studies: 3
       Study label: author
        Study size: _meta_studysize
      Summary data: sepsis_yes_inmfr sepsis_no_inmfr sepsis_yes_nonmfr sepsis_no_nonmfr

       Effect size
              Type: lnoratio
             Label: Log odds-ratio
          Variable: _meta_es
   Zero-cells adj.: 0.5, only0

         Precision
         Std. err.: _meta_se
                CI: [_meta_cil, _meta_ciu]
          CI level: 95%

  Model and method
             Model: Random effects
            Method: REML
```

```
. meta summarize, random(reml) eform

  Effect-size label: Log odds-ratio
        Effect size: _meta_es
          Std. err.: _meta_se
        Study label: author

Meta-analysis summary                     Number of studies =      3
Random-effects model                      Heterogeneity:
Method: REML                                          tau2 =  0.0000
                                                    I2 (%) =    0.00
                                                        H2 =    1.00

--------------------------------------------------------------------
           Study |     Odds ratio    [95% conf. interval]  % weight
-----------------+--------------------------------------------------
           Gause |          1.468       0.055      38.914     10.80
           Coles |          1.042       0.296       3.671     73.10
             Lee |          1.000       0.068      14.640     16.10
-----------------+--------------------------------------------------
      exp(theta) |          1.074       0.366       3.153
--------------------------------------------------------------------
Test of theta = 0: z = 0.13                      Prob > |z| = 0.8968
Test of homogeneity: Q = chi2(2) = 0.04            Prob > Q = 0.9802
```

```
. meta forestplot, random(reml) esrefline nullrefline eform

  Effect-size label: Log odds-ratio
        Effect size: _meta_es
          Std. err.: _meta_se
        Study label: author
```

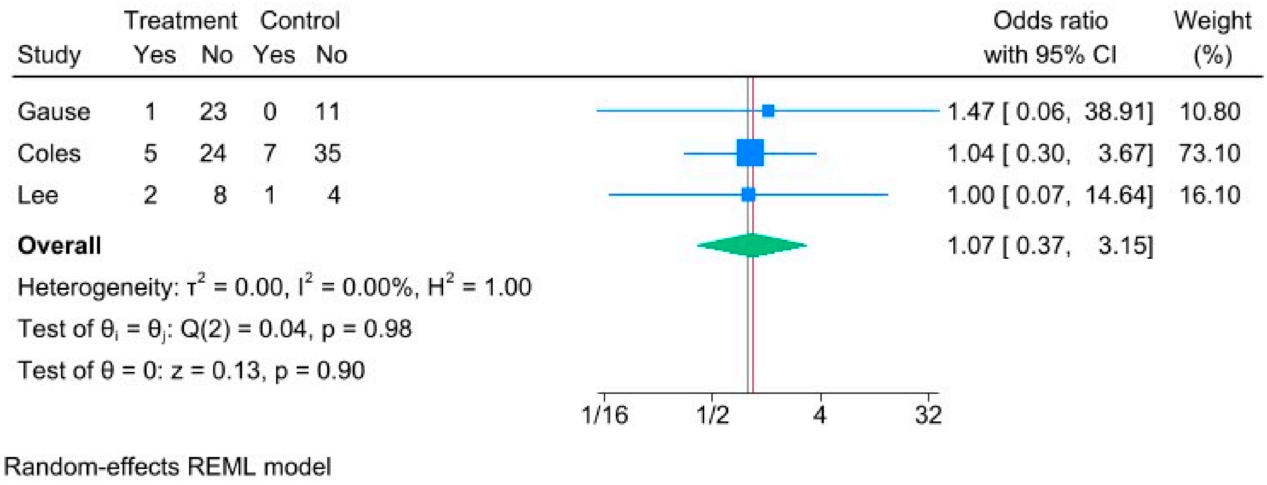


```
. meta funnelplot

  Effect-size label: Log odds-ratio
        Effect size: _meta_es
          Std. err.: _meta_se
              Model: Common effect
             Method: Inverse-variance
```

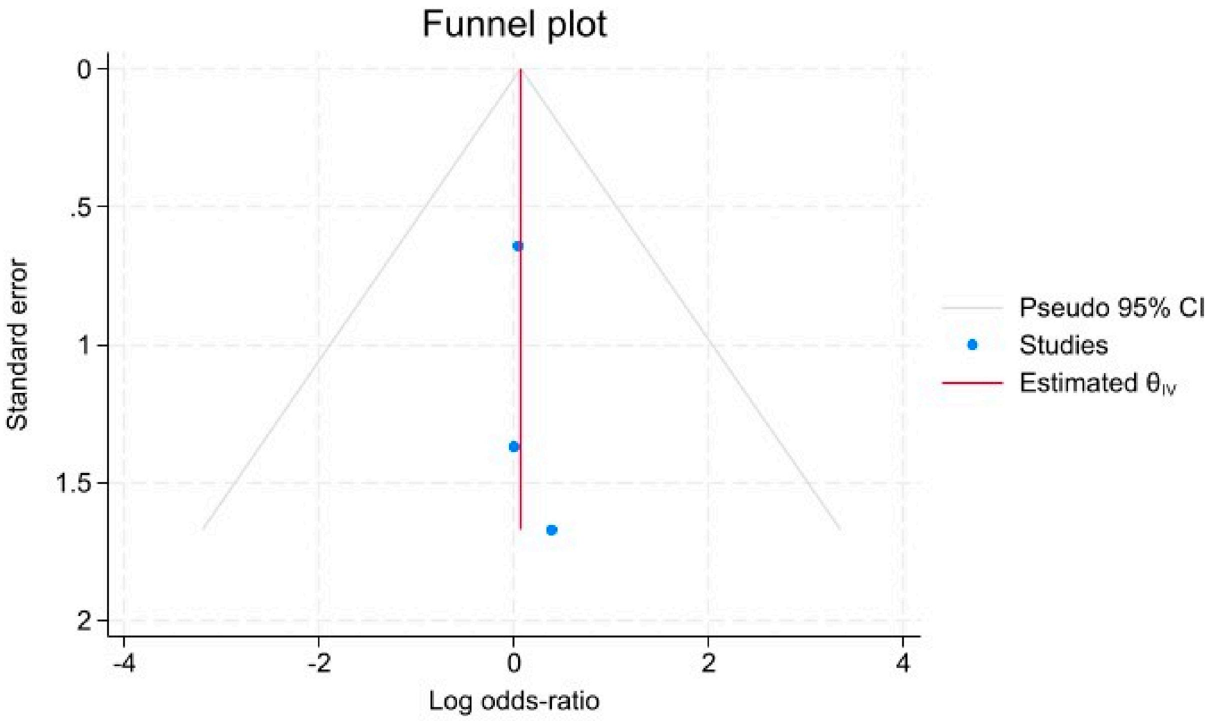


```
. meta bias, egger

  Effect-size label: Log odds-ratio
        Effect size: _meta_es
          Std. err.: _meta_se

Regression-based Egger test for small-study effects
Random-effects model
Method: REML

H0: beta1 = 0; no small-study effects
            beta1 =      0.18
      SE of beta1 =     1.431
```

```
              z =       0.13
      Prob > |z| =    0.9004
```

```
. meta labbeplot, random(reml)

  Effect-size label: Log odds-ratio
        Effect size: _meta_es
          Std. err.: _meta_se
       Summary data: sepsis_yes_inmfr sepsis_no_inmfr sepsis_yes_nonmfr sepsis_no_nonmfr
              Model: Random effects
             Method: REML
```

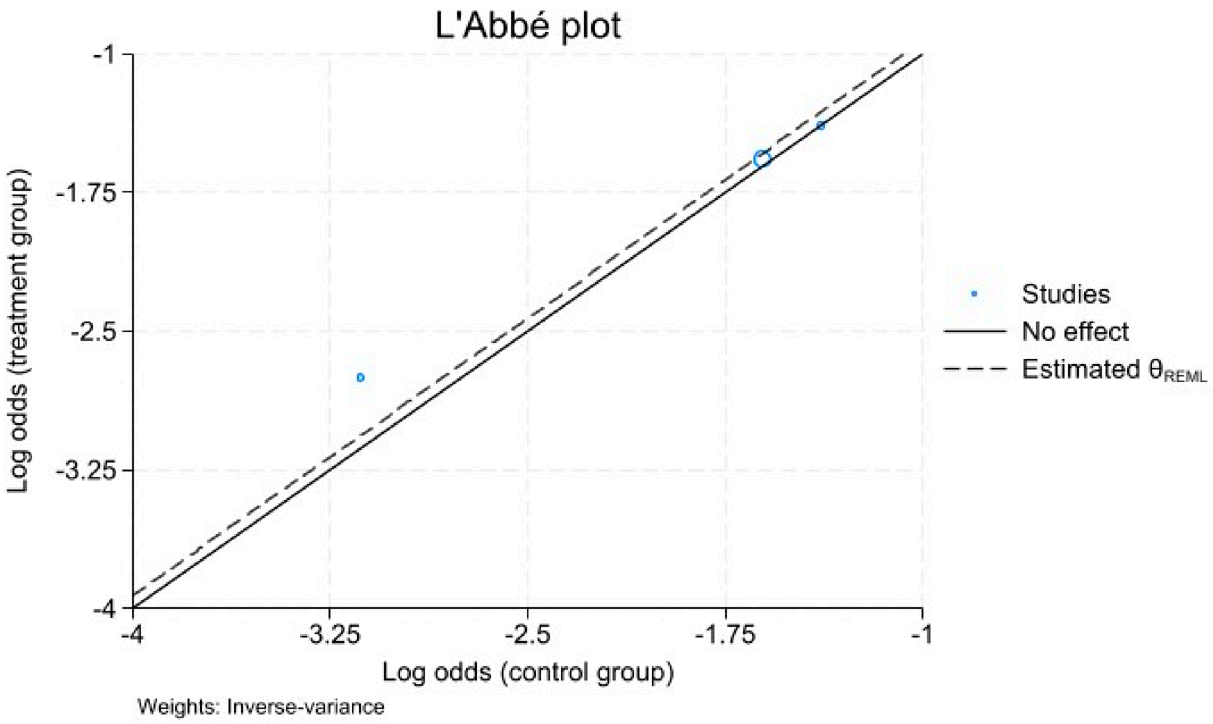


```
. meta galbraith, random(reml)

  Effect-size label: Log odds-ratio
        Effect size: _meta_es
          Std. err.: _meta_se
              Model: Random effects
             Method: REML
```

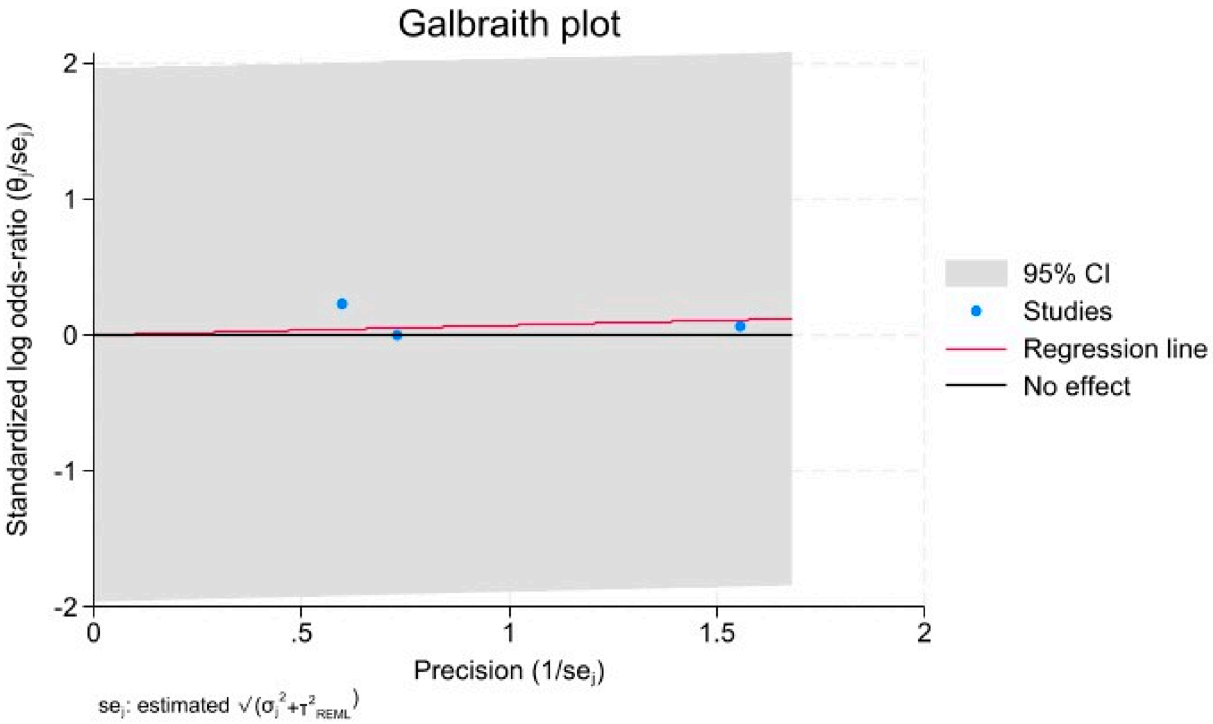


**FIXED-EFFECTS MODEL**

```
. meta summarize, fixed(ivariance) eform

  Effect-size label: Log odds-ratio
        Effect size: _meta_es
          Std. err.: _meta_se
        Study label: author

Meta-analysis summary                     Number of studies =      3
Fixed-effects model                       Heterogeneity:
Method: Inverse-variance                            I2 (%) =    0.00
                                                        H2 =    1.00

--------------------------------------------------------------------
            Study |     Odds ratio    [95% conf. interval]  % weight
------------------+-------------------------------------------------
            Gause |          1.468       0.055     38.914     10.80
            Coles |          1.042       0.296      3.671     73.10
              Lee |          1.000       0.068     14.640     16.10
------------------+-------------------------------------------------
       exp(theta) |          1.074       0.366      3.153
--------------------------------------------------------------------
Test of theta = 0: z = 0.13                       Prob > |z| = 0.8968
Test of homogeneity: Q = chi2(2) = 0.04             Prob > Q = 0.9802


. meta forestplot, fixed(ivariance) esrefline nullrefline eform

  Effect-size label: Log odds-ratio
        Effect size: _meta_es
          Std. err.: _meta_se
        Study label: author
```

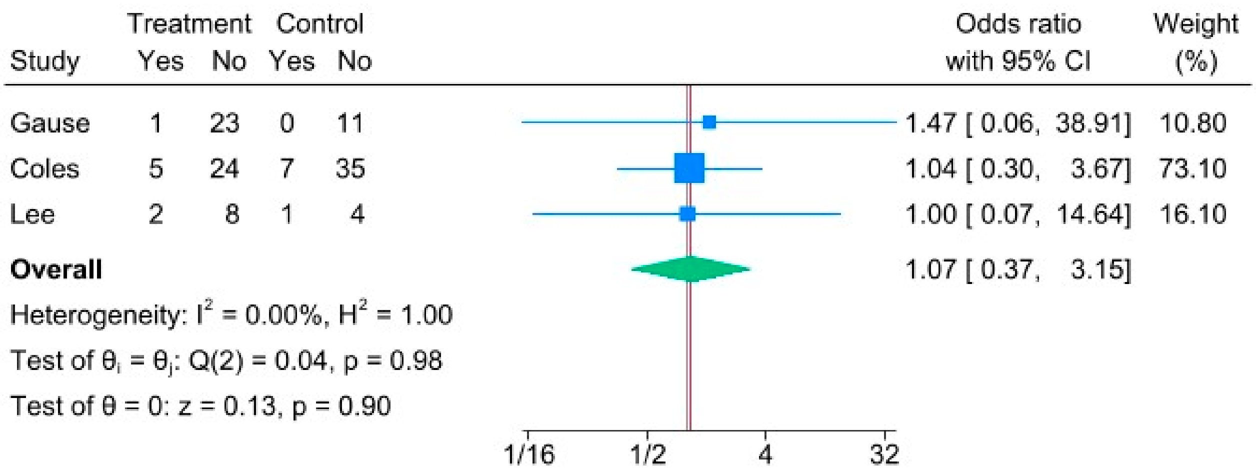


Fixed-effects inverse-variance model

```
. meta funnelplot

  Effect-size label: Log odds-ratio
        Effect size: _meta_es
          Std. err.: _meta_se
              Model: Common effect
             Method: Inverse-variance
```

*Graph overlapping with that obtained with the random-effects model*

```
. meta bias, egger

  Effect-size label: Log odds-ratio
        Effect size: _meta_es
          Std. err.: _meta_se

Regression-based Egger test for small-study effects
Random-effects model
Method: REML

H0: beta1 = 0; no small-study effects
            beta1 =      0.18
      SE of beta1 =     1.431
                z =      0.13
       Prob > |z| =    0.9004


. meta labbeplot, fixed(ivariance)

  Effect-size label: Log odds-ratio
        Effect size: _meta_es
          Std. err.: _meta_se
       Summary data: sepsis_yes_inmfr sepsis_no_inmfr sepsis_yes_nonmfr sepsis_no_nonmfr
              Model: Fixed effects
             Method: Inverse-variance
```

*Graph overlapping with that obtained with the random-effects model*

**TIME FOR RESTORATION OF BOWEL CONTINUITY**
**RANDOM-EFFECTS MODEL**

```
Meta-analysis setting information

 Study information
    No. of studies: 6
       Study label: author
        Study size: _meta_studysize
      Summary data: n_treat mean_treat sd_treat n_control mean_control sd_control

       Effect size
              Type: hedgesg
             Label: Hedges's g
          Variable: _meta_es
   Bias correction: Approximate

         Precision
         Std. err.: _meta_se
    Std. err. adj.: None
                CI: [_meta_cil, _meta_ciu]
          CI level: 95%

  Model and method
             Model: Random effects
            Method: REML
```

**. meta summarize, random(reml)**

```
  Effect-size label: Hedges's g
        Effect size: _meta_es
          Std. err.: _meta_se
        Study label: author

Meta-analysis summary                     Number of studies =      6
Random-effects model                      Heterogeneity:
Method: REML                                          tau2 =  0.0000
                                                    I2 (%) =    0.00
                                                        H2 =    1.00
```

| Study | Hedges's g | [95% conf. interval] | | % weight |
|---|---|---|---|---|
| Gause | 0.046 | -0.730 | 0.821 | 11.70 |
| Yabe | -0.786 | -1.781 | 0.209 | 7.10 |
| Woods (NEC) | 0.043 | -0.414 | 0.500 | 33.63 |
| Woods (SBA) | -0.042 | -0.926 | 0.842 | 9.01 |
| Coles | 0.195 | -0.274 | 0.664 | 31.95 |
| Lee | 0.565 | -0.465 | 1.596 | 6.62 |
| theta | 0.060 | -0.205 | 0.325 | |

```
Test of theta = 0: z = 0.44                   Prob > |z| = 0.6577
Test of homogeneity: Q = chi2(5) = 4.08         Prob > Q = 0.5380
```

**. meta forestplot, random(reml) esrefline nullrefline**

```
  Effect-size label: Hedges's g
        Effect size: _meta_es
          Std. err.: _meta_se
        Study label: author
```

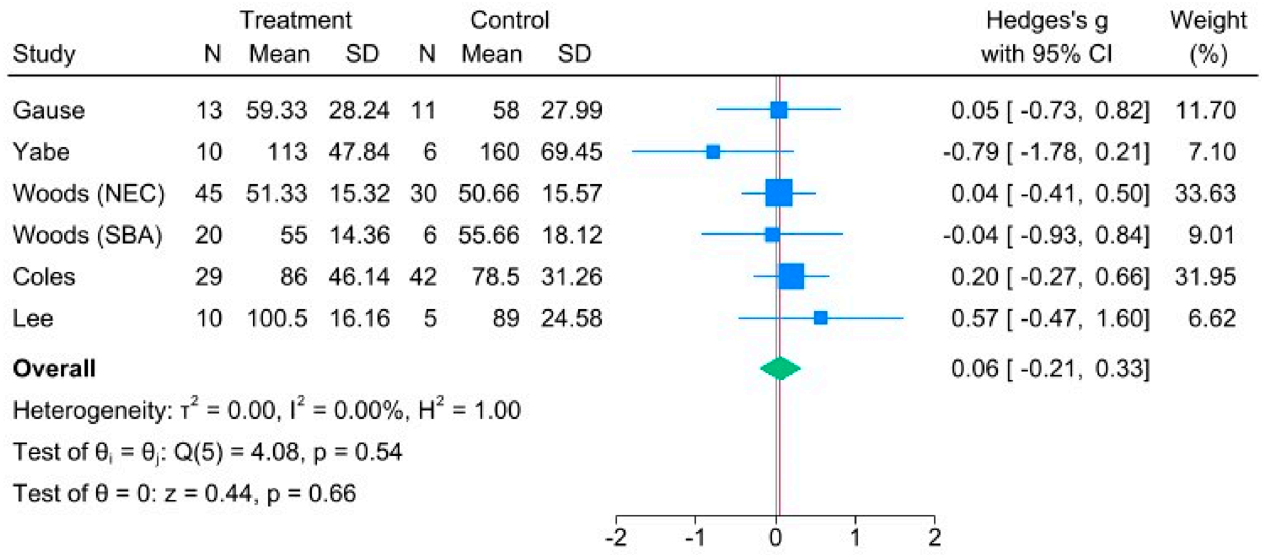


Random-effects REML model

```
. meta funnelplot

  Effect-size label: Hedges's g
        Effect size: _meta_es
          Std. err.: _meta_se
              Model: Common effect
             Method: Inverse-variance
```

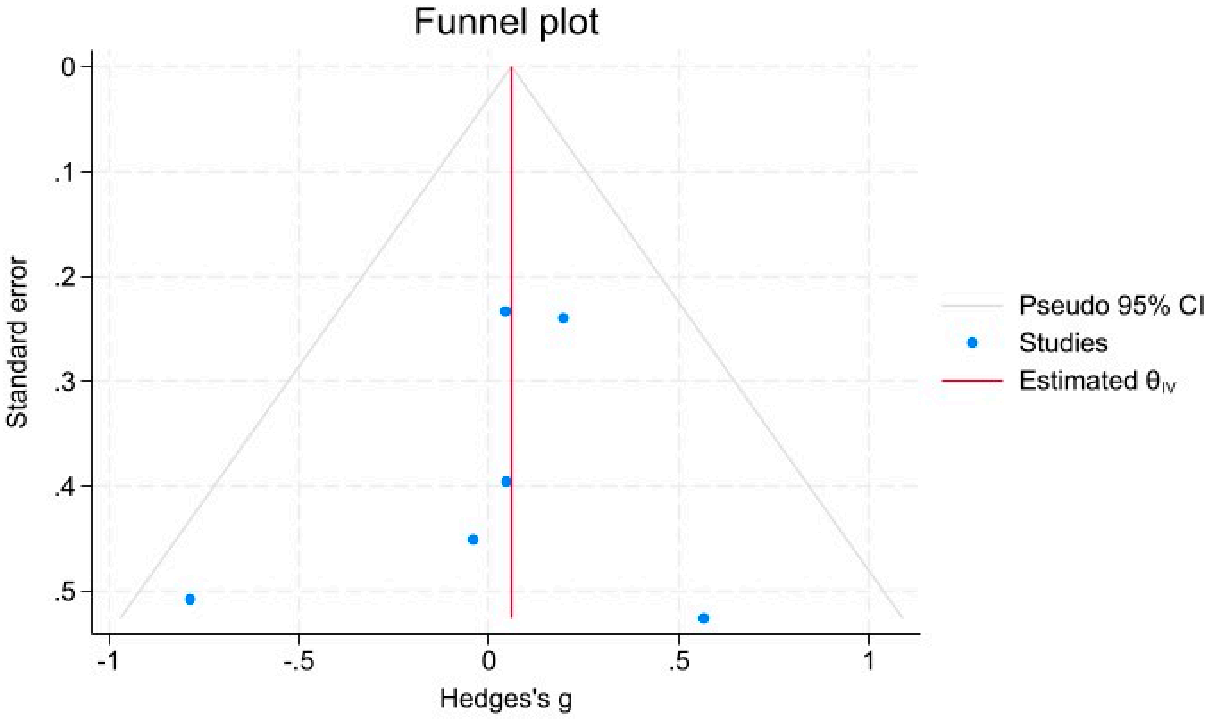


```
. meta bias, egger

  Effect-size label: Hedges's g
        Effect size: _meta_es
          Std. err.: _meta_se

Regression-based Egger test for small-study effects
Random-effects model
Method: REML
```

```
H0: beta1 = 0; no small-study effects
            beta1 =      -0.72
      SE of beta1 =      1.232
                z =      -0.59
       Prob > |z| =     0.5577
```

**. meta galbraith, random(reml)**

```
  Effect-size label: Hedges's g
        Effect size: _meta_es
          Std. err.: _meta_se
              Model: Random effects
             Method: REML
```

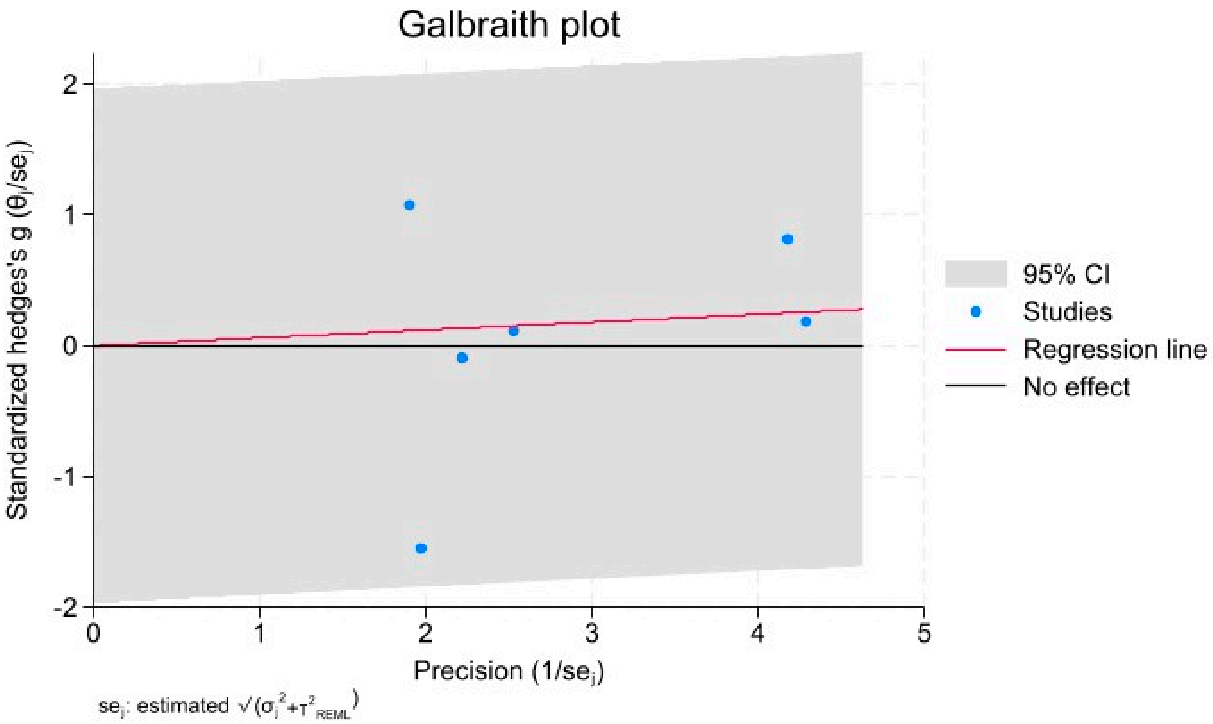


**FIXED-EFFECTS MODEL**

**. meta summarize, fixed(ivariance)**

```
  Effect-size label: Hedges's g
        Effect size: _meta_es
          Std. err.: _meta_se
        Study label: author

Meta-analysis summary                     Number of studies =      6
Fixed-effects model                       Heterogeneity:
Method: Inverse-variance                             I2 (%) =   0.00
                                                         H2 =   1.00
```

| Study | Hedges's g | [95% conf. | interval] | % weight |
|---|---|---|---|---|
| Gause | 0.046 | -0.730 | 0.821 | 11.70 |
| Yabe | -0.786 | -1.781 | 0.209 | 7.10 |
| Woods (NEC) | 0.043 | -0.414 | 0.500 | 33.63 |
| Woods (SBA) | -0.042 | -0.926 | 0.842 | 9.01 |
| Coles | 0.195 | -0.274 | 0.664 | 31.95 |
| Lee | 0.565 | -0.465 | 1.596 | 6.62 |
| theta | 0.060 | -0.205 | 0.325 | |

```
Test of theta = 0: z = 0.44                     Prob > |z| = 0.6577
Test of homogeneity: Q = chi2(5) = 4.08           Prob > Q = 0.5380
```

**. meta forestplot, fixed(ivariance) esrefline nullrefline**

```
  Effect-size label: Hedges's g
        Effect size: _meta_es
          Std. err.: _meta_se
        Study label: author
```

| Study | Treatment N | Treatment Mean | Treatment SD | Control N | Control Mean | Control SD | Hedges's g with 95% CI | Weight (%) |
|---|---|---|---|---|---|---|---|---|
| Gause | 13 | 59.33 | 28.24 | 11 | 58 | 27.99 | 0.05 [ -0.73, 0.82] | 11.70 |
| Yabe | 10 | 113 | 47.84 | 6 | 160 | 69.45 | -0.79 [ -1.78, 0.21] | 7.10 |
| Woods (NEC) | 45 | 51.33 | 15.32 | 30 | 50.66 | 15.57 | 0.04 [ -0.41, 0.50] | 33.63 |
| Woods (SBA) | 20 | 55 | 14.36 | 6 | 55.66 | 18.12 | -0.04 [ -0.93, 0.84] | 9.01 |
| Coles | 29 | 86 | 46.14 | 42 | 78.5 | 31.26 | 0.20 [ -0.27, 0.66] | 31.95 |
| Lee | 10 | 100.5 | 16.16 | 5 | 89 | 24.58 | 0.57 [ -0.47, 1.60] | 6.62 |
| **Overall** | | | | | | | 0.06 [ -0.21, 0.33] | |

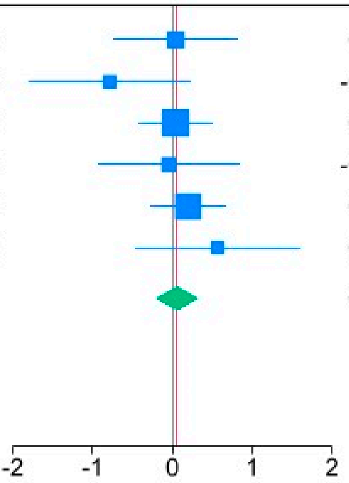


Heterogeneity: $I^2 = 0.00\%$, $H^2 = 1.00$

Test of $\theta_i = \theta_j$: Q(5) = 4.08, p = 0.54

Test of $\theta = 0$: z = 0.44, p = 0.66

Fixed-effects inverse-variance model

```
. meta bias, egger

  Effect-size label: Hedges's g
        Effect size: _meta_es
          Std. err.: _meta_se

Regression-based Egger test for small-study effects
Random-effects model
Method: REML

H0: beta1 = 0; no small-study effects
            beta1 =      -0.72
      SE of beta1 =      1.232
                z =      -0.59
       Prob > |z| =     0.5577
```

**MEAN PEAK BILIRUBIN**
**RANDOM-EFFECTS MODEL**

```
Meta-analysis setting information

 Study information
    No. of studies: 4
       Study label: author
        Study size: _meta_studysize
      Summary data: n_treat mean_treat sd_treat n_control mean_control sd_control

       Effect size
              Type: hedgesg
             Label: Hedges's g
          Variable: _meta_es
   Bias correction: Approximate

         Precision
         Std. err.: _meta_se
    Std. err. adj.: None
                CI: [_meta_cil, _meta_ciu]
          CI level: 95%

  Model and method
             Model: Random effects
            Method: REML
```

**. meta summarize, random(reml)**

```
  Effect-size label: Hedges's g
        Effect size: _meta_es
          Std. err.: _meta_se
        Study label: author

Meta-analysis summary                     Number of studies =      4
Random-effects model                      Heterogeneity:
Method: REML                                           tau2 =  3.6340
                                                     I2 (%) =   96.27
                                                         H2 =   26.84

--------------------------------------------------------------------
            Study |     Hedges's g    [95% conf. interval]  % weight
------------------+-------------------------------------------------
              Lau |         -4.687      -5.559      -3.816     24.75
            Gause |         -0.606      -1.400       0.188     24.97
      Woods (NEC) |         -0.811      -1.286      -0.335     25.68
      Woods (SBA) |         -0.906      -1.823       0.012     24.61
------------------+-------------------------------------------------
            theta |         -1.742      -3.651       0.166
--------------------------------------------------------------------
Test of theta = 0: z = -1.79                     Prob > |z| = 0.0736
Test of homogeneity: Q = chi2(3) = 65.50           Prob > Q = 0.0000
```

**. meta forestplot, random(reml) esrefline nullrefline**

```
  Effect-size label: Hedges's g
        Effect size: _meta_es
          Std. err.: _meta_se
        Study label: author
```

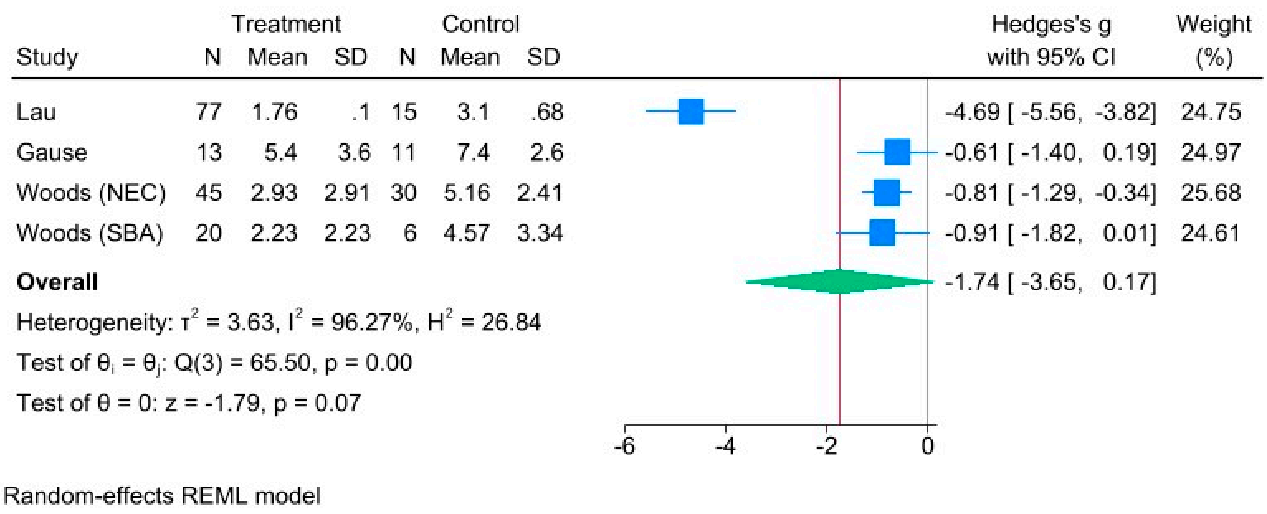


```
. meta funnelplot

  Effect-size label: Hedges's g
        Effect size: _meta_es
          Std. err.: _meta_se
              Model: Common effect
             Method: Inverse-variance
```

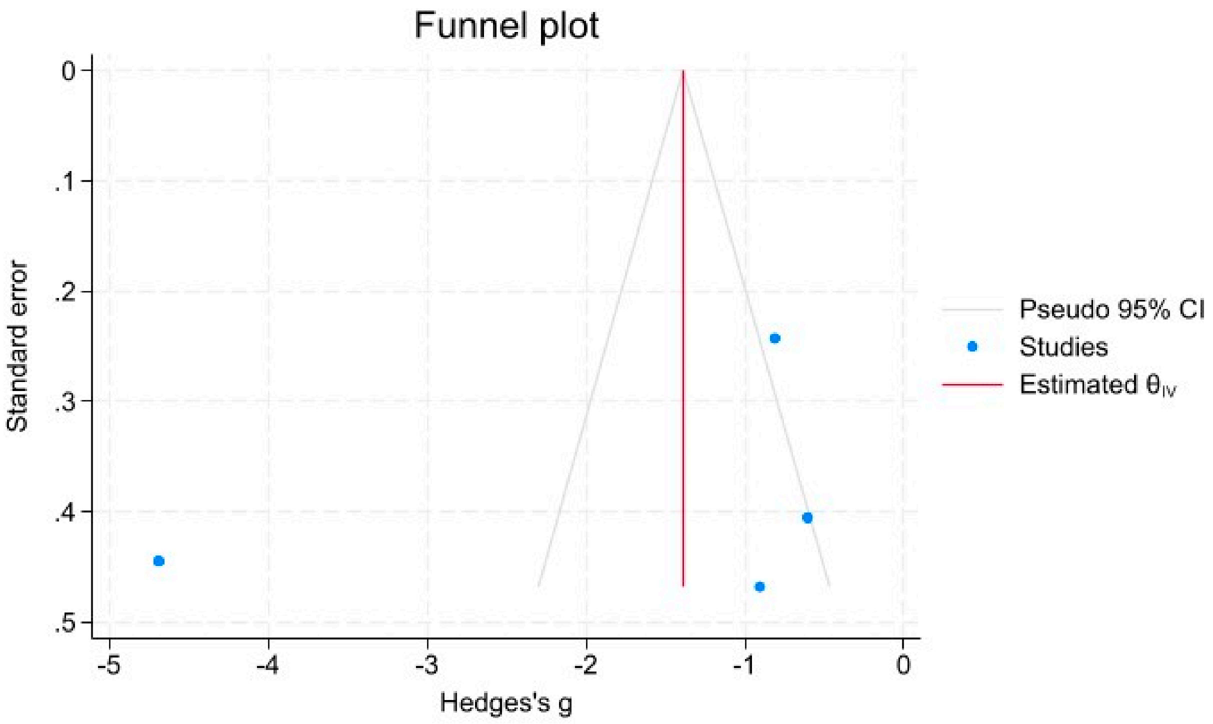


```
. meta bias, egger

  Effect-size label: Hedges's g
        Effect size: _meta_es
          Std. err.: _meta_se

Regression-based Egger test for small-study effects
Random-effects model
Method: REML

H0: beta1 = 0; no small-study effects
            beta1 =     -6.96
      SE of beta1 =    12.577
```

```
              z =     -0.55
       Prob > |z| =    0.5803
```

```
. meta galbraith, random(reml)

  Effect-size label: Hedges's g
        Effect size: _meta_es
          Std. err.: _meta_se
              Model: Random effects
             Method: REML
```

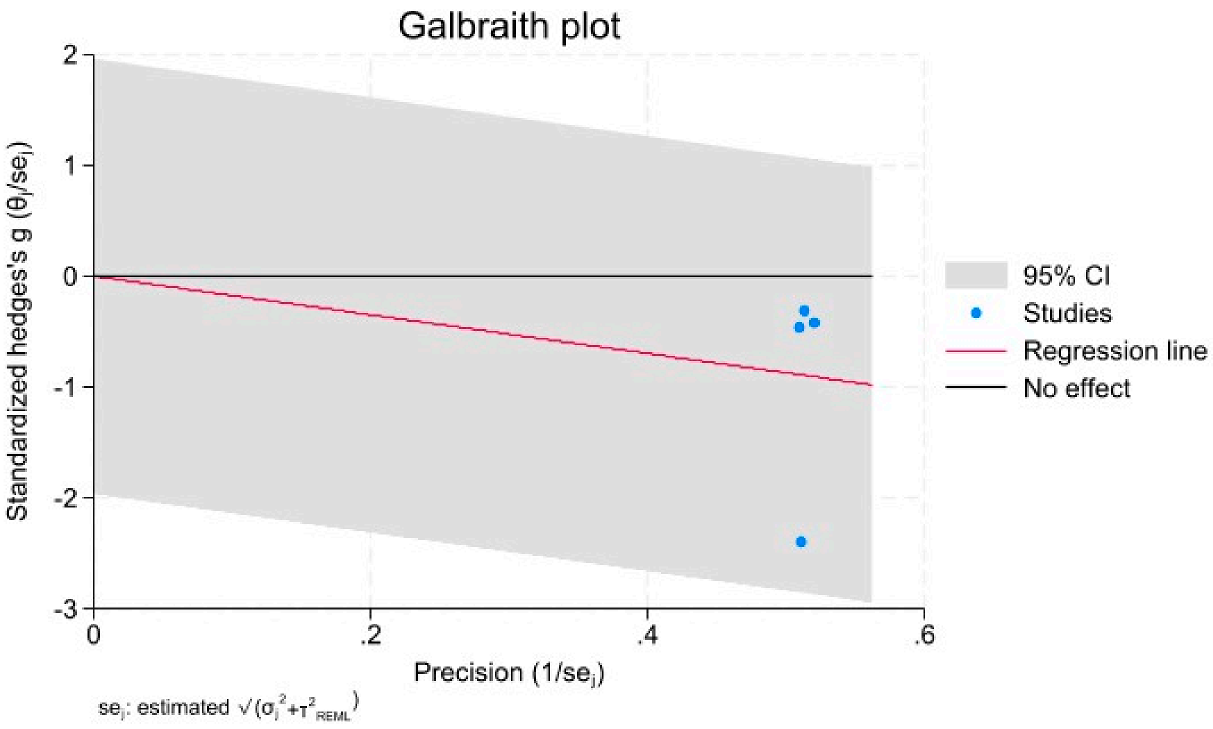


**FIXED-EFFECTS MODEL**

```
. meta summarize, fixed(ivariance)

  Effect-size label: Hedges's g
        Effect size: _meta_es
          Std. err.: _meta_se
        Study label: author

Meta-analysis summary                     Number of studies =      4
Fixed-effects model                       Heterogeneity:
Method: Inverse-variance                             I2 (%) =   95.42
                                                         H2 =   21.83

--------------------------------------------------------------------
            Study |     Hedges's g    [95% conf. interval]  % weight
------------------+-------------------------------------------------
              Lau |         -4.687      -5.559      -3.816     15.45
            Gause |         -0.606      -1.400       0.188     18.62
      Woods (NEC) |         -0.811      -1.286      -0.335     51.97
      Woods (SBA) |         -0.906      -1.823       0.012     13.96
------------------+-------------------------------------------------
            theta |         -1.385      -1.728      -1.042
--------------------------------------------------------------------
Test of theta = 0: z = -7.92                     Prob > |z| = 0.0000
Test of homogeneity: Q = chi2(3) = 65.50           Prob > Q = 0.0000
```

```
. meta forestplot, fixed(ivariance) esrefline nullrefline

  Effect-size label: Hedges's g
        Effect size: _meta_es
          Std. err.: _meta_se
        Study label: author
```

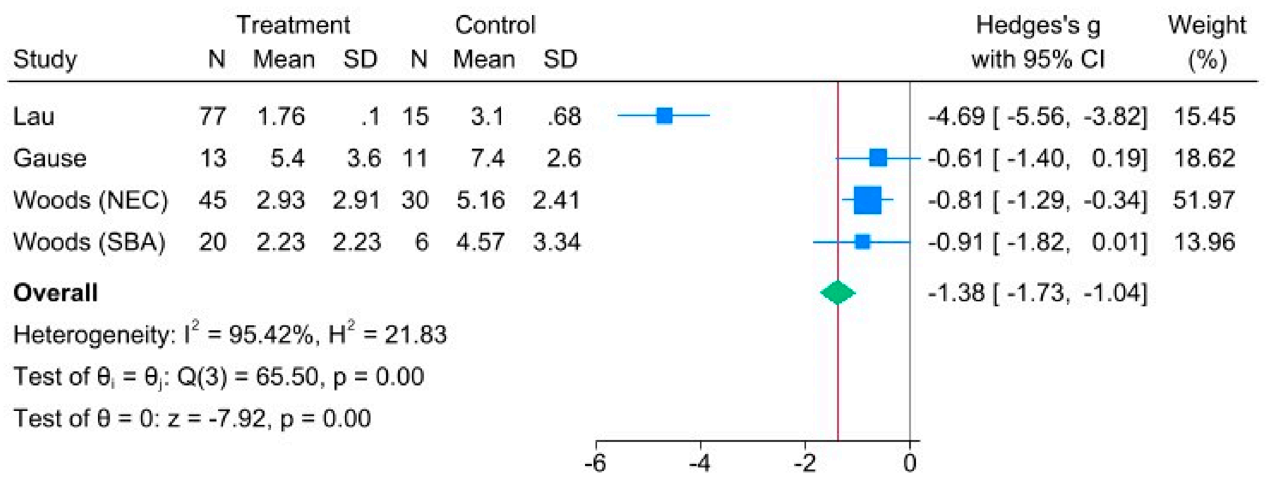


Fixed-effects inverse-variance model

```
. meta funnelplot

  Effect-size label: Hedges's g
        Effect size: _meta_es
          Std. err.: _meta_se
              Model: Common effect
             Method: Inverse-variance
```

*Graph overlapping with that obtained with the random-effects model*

```
. meta bias, egger

  Effect-size label: Hedges's g
        Effect size: _meta_es
          Std. err.: _meta_se

Regression-based Egger test for small-study effects
Random-effects model
Method: REML

H0: beta1 = 0; no small-study effects
            beta1 =     -6.96
      SE of beta1 =    12.577
                z =     -0.55
       Prob > |z| =    0.5803
```

**DURATION PARENTERAL NUTRITION**

**RANDOM-EFFECTS MODEL**

```
Meta-analysis setting information

 Study information
    No. of studies: 6
       Study label: author
        Study size: _meta_studysize
      Summary data: n_treat mean_treat sd_treat n_control mean_control sd_control

       Effect size
              Type: hedgesg
             Label: Hedges's g
          Variable: _meta_es
   Bias correction: Approximate

         Precision
         Std. err.: _meta_se
    Std. err. adj.: None
                CI: [_meta_cil, _meta_ciu]
          CI level: 95%

  Model and method
             Model: Random effects
            Method: REML
```

**. meta summarize, random(reml)**

```
  Effect-size label: Hedges's g
        Effect size: _meta_es
          Std. err.: _meta_se
        Study label: author

Meta-analysis summary                     Number of studies =      6
Random-effects model                      Heterogeneity:
Method: REML                                          tau2 =  1.8192
                                                    I2 (%) =   91.88
                                                        H2 =   12.32

--------------------------------------------------------------------
           Study |     Hedges's g    [95% conf. interval]  % weight
-----------------+--------------------------------------------------
             Lau |         -3.695      -4.460      -2.929     17.04
           Gause |         -0.781      -1.588       0.025     16.89
            Yabe |         -1.918      -3.083      -0.753     15.46
     Woods (NEC) |         -0.557      -1.023      -0.091     17.91
     Woods (SBA) |         -0.333      -1.221       0.555     16.59
             Lee |          0.159      -0.853       1.171     16.11
-----------------+--------------------------------------------------
           theta |         -1.187      -2.323      -0.051
--------------------------------------------------------------------
Test of theta = 0: z = -2.05                    Prob > |z| = 0.0405
Test of homogeneity: Q = chi2(5) = 60.66          Prob > Q = 0.0000
```

**. meta forestplot, random(reml) esrefline nullrefline**

```
  Effect-size label: Hedges's g
        Effect size: _meta_es
          Std. err.: _meta_se
        Study label: author
```

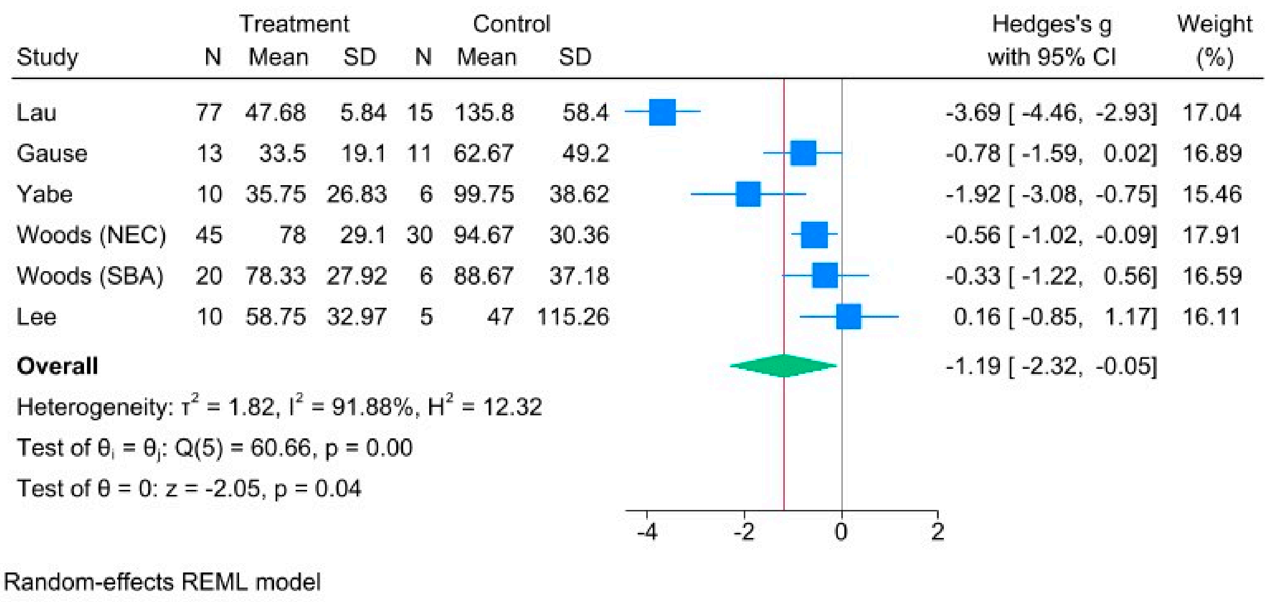


```
. meta funnelplot

  Effect-size label: Hedges's g
        Effect size: _meta_es
          Std. err.: _meta_se
              Model: Common effect
             Method: Inverse-variance
```

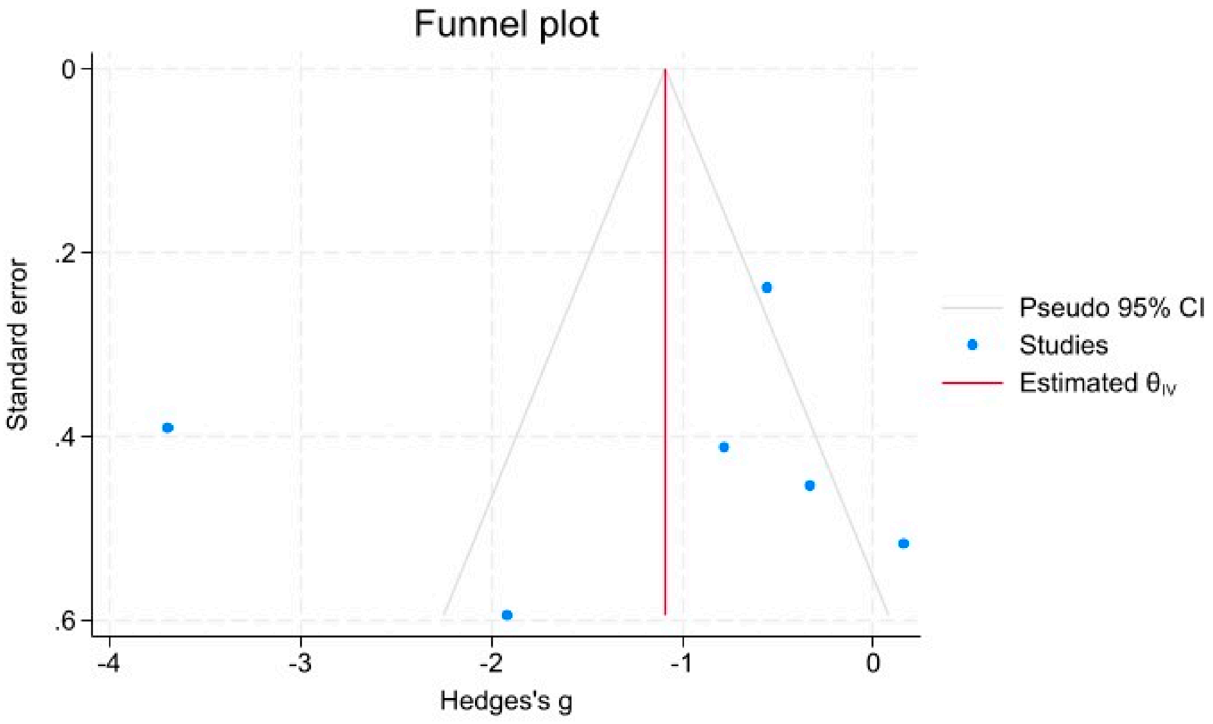


```
. meta bias, egger

  Effect-size label: Hedges's g
        Effect size: _meta_es
          Std. err.: _meta_se

Regression-based Egger test for small-study effects
Random-effects model
Method: REML

H0: beta1 = 0; no small-study effects
            beta1 =     -0.21
```

```
      SE of beta1 =      5.818
                z =      -0.04
       Prob > |z| =     0.9712

. meta galbraith, random(reml)

  Effect-size label: Hedges's g
        Effect size: _meta_es
          Std. err.: _meta_se
              Model: Random effects
             Method: REML
```

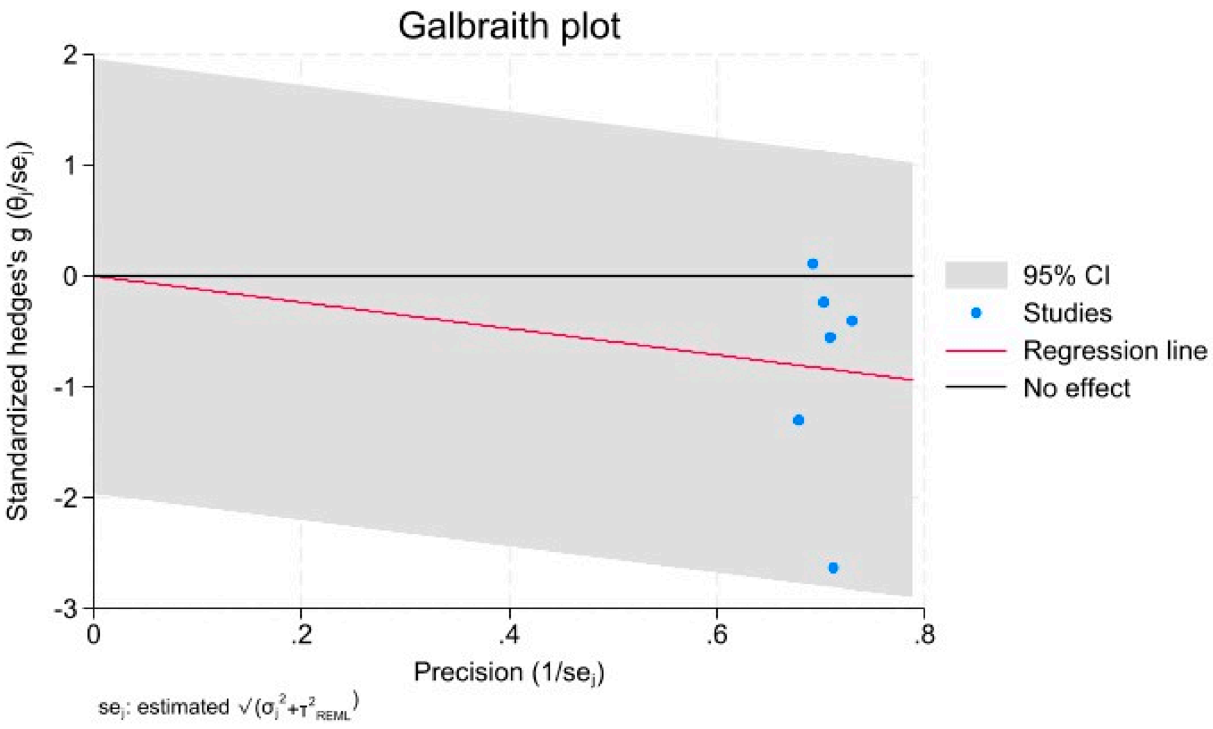


**FIXED-EFFECTS MODEL**

```
. meta summarize, fixed(ivariance)

  Effect-size label: Hedges's g
        Effect size: _meta_es
          Std. err.: _meta_se
        Study label: author

Meta-analysis summary                     Number of studies =      6
Fixed-effects model                       Heterogeneity:
Method: Inverse-variance                             I2 (%) =   91.76
                                                         H2 =   12.13

--------------------------------------------------------------------
            Study |    Hedges's g    [95% conf. interval]  % weight
------------------+-------------------------------------------------
              Lau |        -3.695      -4.460      -2.929     15.75
            Gause |        -0.781      -1.588       0.025     14.20
             Yabe |        -1.918      -3.083      -0.753      6.80
     Woods (NEC) |        -0.557      -1.023      -0.091     42.53
     Woods (SBA) |        -0.333      -1.221       0.555     11.70
              Lee |         0.159      -0.853       1.171      9.01
------------------+-------------------------------------------------
            theta |        -1.085      -1.389      -0.781
--------------------------------------------------------------------
Test of theta = 0: z = -7.00                        Prob > |z| = 0.0000
Test of homogeneity: Q = chi2(5) = 60.66              Prob > Q = 0.0000

. meta forestplot, fixed(ivariance) esrefline nullrefline

  Effect-size label: Hedges's g
        Effect size: _meta_es
```

```
        Std. err.: _meta_se
      Study label: author
```

| Study | Treatment N | Treatment Mean | Treatment SD | Control N | Control Mean | Control SD | Hedges's g with 95% CI | Weight (%) |
|---|---|---|---|---|---|---|---|---|
| Lau | 77 | 47.68 | 5.84 | 15 | 135.8 | 58.4 | -3.69 [ -4.46, -2.93] | 15.75 |
| Gause | 13 | 33.5 | 19.1 | 11 | 62.67 | 49.2 | -0.78 [ -1.59, 0.02] | 14.20 |
| Yabe | 10 | 35.75 | 26.83 | 6 | 99.75 | 38.62 | -1.92 [ -3.08, -0.75] | 6.80 |
| Woods (NEC) | 45 | 78 | 29.1 | 30 | 94.67 | 30.36 | -0.56 [ -1.02, -0.09] | 42.53 |
| Woods (SBA) | 20 | 78.33 | 27.92 | 6 | 88.67 | 37.18 | -0.33 [ -1.22, 0.56] | 11.70 |
| Lee | 10 | 58.75 | 32.97 | 5 | 47 | 115.26 | 0.16 [ -0.85, 1.17] | 9.01 |
| **Overall** | | | | | | | -1.09 [ -1.39, -0.78] | |

Heterogeneity: $I^2$ = 91.76%, $H^2$ = 12.13

Test of $\theta_i = \theta_j$: Q(5) = 60.66, p = 0.00

Test of θ = 0: z = -7.00, p = 0.00

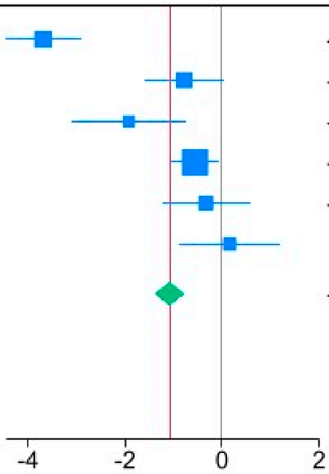


Fixed-effects inverse-variance model

```
. meta funnelplot

  Effect-size label: Hedges's g
        Effect size: _meta_es
          Std. err.: _meta_se
              Model: Common effect
             Method: Inverse-variance


. meta bias, egger

  Effect-size label: Hedges's g
        Effect size: _meta_es
          Std. err.: _meta_se

Regression-based Egger test for small-study effects
Random-effects model
Method: REML

H0: beta1 = 0; no small-study effects
            beta1 =      -0.21
      SE of beta1 =      5.818
                z =      -0.04
       Prob > |z| =     0.9712
```

**TOTAL LENGTH OF NICU STAY**

```
. ttesti 77 125.7 20.18 15 200.2 63.28, unequal

Two-sample t test with unequal variances
------------------------------------------------------------------------------
         |     Obs        Mean    Std. Err.   Std. Dev.   [95% Conf. Interval]
---------+--------------------------------------------------------------------
       x |      77       125.7    2.299724       20.18    121.1197    130.2803
       y |      15       200.2    16.33883       63.28    165.1567    235.2433
---------+--------------------------------------------------------------------
combined |      92    137.8467    4.326201    41.49546    129.2533    146.4402
---------+--------------------------------------------------------------------
    diff |               -74.5    16.49988               -109.7616   -39.23837
------------------------------------------------------------------------------
    diff = mean(x) - mean(y)                                      t =  -4.5152
Ho: diff = 0                     Satterthwaite's degrees of freedom =  14.5592
    Ha: diff < 0                 Ha: diff != 0                 Ha: diff > 0
 Pr(T < t) = 0.0002         Pr(|T| > |t|) = 0.0004          Pr(T > t) = 0.9998
```

**TOTAL LENGTH OF HOSPITAL STAY**

```
. ttesti 77 179.3 33.09 15 262.9 95, unequal

Two-sample t test with unequal variances
------------------------------------------------------------------------------
         |     Obs        Mean    Std. Err.   Std. Dev.   [95% Conf. Interval]
---------+--------------------------------------------------------------------
       x |      77       179.3    3.770955       33.09    171.7895    186.8105
       y |      15       262.9    24.52889          95    210.2908    315.5092
---------+--------------------------------------------------------------------
combined |      92    192.9304    5.959209    57.15872    181.0932    204.7677
---------+--------------------------------------------------------------------
    diff |               -83.6    24.81707               -136.6008   -30.59923
------------------------------------------------------------------------------
    diff = mean(x) - mean(y)                                      t =  -3.3686
Ho: diff = 0                     Satterthwaite's degrees of freedom =  14.6681
    Ha: diff < 0                 Ha: diff != 0                 Ha: diff > 0
 Pr(T < t) = 0.0022         Pr(|T| > |t|) = 0.0043          Pr(T > t) = 0.9978
```

**DISCREPANCY BETWEEN THE STOMA CALIBERS**

```
. cci 19 58 8 7, exact
                                                         Proportion
                 |   Exposed   Unexposed  |      Total      exposed
-----------------+------------------------+------------------------
           Cases |        19          58  |         77       0.2468
        Controls |         8           7  |         15       0.5333
-----------------+------------------------+------------------------
           Total |        27          65  |         92       0.2935
                 |                        |
                 |      Point estimate    |    [95% Conf. Interval]
                 |------------------------+------------------------
      Odds ratio |         .2866379       |    .0780851    1.053655 (exact)
 Prev. frac. ex. |         .7133621       |   -.053655    .9219149 (exact)
 Prev. frac. pop |         .3804598       |
                 +-------------------------------------------------
                                  1-sided Fisher's exact P = 0.0307
                                  2-sided Fisher's exact P = 0.0339
```

**POST-RESTORATION OF BOWEL CONTINUITY COMPLICATIONS**

```
. cci 2 3 75 12, exact
                                                         Proportion
                 |   Exposed   Unexposed  |      Total     Exposed
-----------------+------------------------+------------------------
           Cases |         2           3  |          5       0.4000
        Controls |        75          12  |         87       0.8621
-----------------+------------------------+------------------------
           Total |        77          15  |         92       0.8370
                 |                        |
                 |      Point estimate    |    [95% Conf. Interval]
                 |------------------------+------------------------
      Odds ratio |         .1066667       |    .0084488    1.071484 (exact)
 Prev. frac. ex. |         .8933333       |   -.0714842    .9915512 (exact)
 Prev. frac. pop |         .7701149       |
                 +-------------------------------------------------
                                  1-sided Fisher's exact P = 0.0293
                                  2-sided Fisher's exact P = 0.0293
```
