# Supplementary material for: Mucous Fistula Refeeding in Newborns: Why, When, How, and Where? Insights from a Systematic Review
Source: Nutrients. 2025 Jul 30;17(15):2490. doi: 10.3390/nu17152490 (PMC12348941; doi:10.3390/nu17152490)
Supplement: Supplementary file 1 [file nutrients-17-02490-s001.zip › Supplementary Material – Table S6.pdf]

Outcome	Number of studies	Number of participants	Random-effects model – Results
Cholestasis	3	245	OR 0.15, 95%CI: 0.07, 0.32, p<0.0001*
Catheter-Related Sepsis	3	121	OR 1.07, 95%CI: 0.37, 3.15, p =0.90 *
Time in RBC	5	227	Hedges's g 0.06, 95%CI: -0.21, 0.33, p=0.66 *
Mean Peak Bilirubin	3	217	Hedges's g -1.74, 95%CI: -3.65, 0.17, p=0.07 *
Duration of PN	5	248	Hedges's g -1.19, 95% CI -2.32,-0.05, p=0.04 *
Total Length of Hospital Stay	1	92	Mean difference -83.6, Std. Err. 24.82, 95%CI -136.60, -30.06, p=0.0043 †
Length of NICU stay	1	92	Mean difference -74.5, Std. Err. 16.50, 95%CI -109.76, -39.24, p=0.0004) †
Reduction in Size Discrepancy	1	92	OR 0.29, 95% CI 0.08, 1.05, p=0.04 §
Anastomosis Complications	1	92	OR 0.11, 95% CI 0.01, 1.07, p=0.03§

Table S6. **Overview of Results**. Mucous Fistula Refeeding (MFR). Restoration of Bowel Continuity (RBC). Neonatal Intensive Care (NICU). Parenteral Nutrition (PN). Legend: *Random-effects model, † t-test, § Fisher's exact test
